# Identifying genetic interactions associated with late-onset Alzheimer’s disease

**DOI:** 10.1186/s13040-014-0035-z

**Published:** 2014-12-19

**Authors:** Charalampos S Floudas, Nara Um, M Ilyas Kamboh, Michael M Barmada, Shyam Visweswaran

**Affiliations:** Department of Biomedical Informatics, University of Pittsburgh, 5607 Baum Boulevard, Pittsburgh, PA 15206 USA; Department of Human Genetics, University of Pittsburgh, 130 De Soto Street, Pittsburgh, PA 15261 USA; The Intelligent Systems Program, University of Pittsburgh, 5113 Sennott Square 210 South Bouquet Street, Pittsburgh, PA 15260 USA

**Keywords:** Genome-wide association study, Epistasis, Alzheimer’s disease, Bayesian networks

## Abstract

**Background:**

Identifying genetic interactions in data obtained from genome-wide association studies (GWASs) can help in understanding the genetic basis of complex diseases. The large number of single nucleotide polymorphisms (SNPs) in GWASs however makes the identification of genetic interactions computationally challenging. We developed the Bayesian Combinatorial Method (BCM) that can identify pairs of SNPs that in combination have high statistical association with disease.

**Results:**

We applied BCM to two late-onset Alzheimer’s disease (LOAD) GWAS datasets to identify SNPs that interact with known Alzheimer associated SNPs. We also compared BCM with logistic regression that is implemented in PLINK. Gene Ontology analysis of genes from the top 200 dataset SNPs for both GWAS datasets showed overrepresentation of LOAD-related terms. Four genes were common to both datasets: *APOE* and *APOC1*, which have well established associations with LOAD, and *CAMK1D* and *FBXL13*, not previously linked to LOAD but having evidence of involvement in LOAD. Supporting evidence was also found for additional genes from the top 30 dataset SNPs.

**Conclusion:**

BCM performed well in identifying several SNPs having evidence of involvement in the pathogenesis of LOAD that would not have been identified by univariate analysis due to small main effect. These results provide support for applying BCM to identify potential genetic variants such as SNPs from high dimensional GWAS datasets.

**Electronic supplementary material:**

The online version of this article (doi:10.1186/s13040-014-0035-z) contains supplementary material, which is available to authorized users.

## Introduction

Elucidating the genetic basis of common diseases will lead to an understanding of the biological mechanisms that underlie such diseases and can help in risk assessment, diagnosis, prognosis and development of new therapies. During the past several decades genetic linkage studies have been effective in mapping genetic loci responsible for many Mendelian diseases that are caused by a single genetic variant [1]. More recently, genetic studies have indicated that most common diseases are likely to be polygenic where multiple genetic variants acting singly and in combination underlie the expression of disease [2].

The commonest type of genetic variation is the single nucleotide polymorphism (SNP) that results when a single nucleotide is replaced by another in the genome sequence. The development of high-throughput genotyping technologies has led to a flurry of genome-wide association studies (GWASs) with the aim of discovering SNPs that are associated with common diseases. GWASs have been moderately successful in identifying SNPs associated with common diseases and traits. However, in most cases the identified SNPs have small effect sizes, and the proportion of heritability explained is quite modest. One view is that SNPs may interact in subtle ways that lead to substantially greater effects than the effect due to any single SNP. Another view is that common diseases may be due to rare and usually deleterious SNPs that cause disease in individual patients and that in different individuals or subpopulations the disease is caused by different deleterious SNPs.

This paper addresses the challenge of identifying interacting SNPs that may have small effects and describes a Bayesian combinatorial method (BCM) for identifying such interactions that are associated with disease. This method has been shown empirically to perform well on low dimensional synthetic data [3]. However, to our knowledge BCM has not been applied to real-world datasets with a large number of SNPs. In this paper we apply BCM to two late-onset Alzheimer’s disease GWAS datasets to identify SNPs that interact with known Alzheimer associated SNPs.

As background, we provide brief summaries about GWASs, genetic interactions, and Alzheimer’s disease in the following sections.

### Genome-wide association studies

The development of high-throughput genotyping technologies that assay hundreds of thousands of SNPs or more, along with the identification of SNPs in the human genome by the International HapMap Project led to the emergence of GWASs. GWASs are typically case–control studies aimed at discovering SNPs – either as disease causing variants or as markers of disease – that are associated with a common disease or trait. The success of GWASs is based in large part on the common disease - common variant hypothesis. This hypothesis posits that common diseases in most individuals are caused by relatively common genetic variants that have low penetrance and hence have small to moderate influence in causing disease. An alternative hypothesis is the common disease - rare variant hypothesis, which posits that many rare variants underlie common diseases and each variant causes disease in relatively few individuals with high penetrance. Both these hypotheses likely contribute to common diseases with genetic variants may range from rare to the common SNPs.

GWAS data is typically analyzed for univariate associations between SNPs and the disease of interest; the statistical tests used include the Pearson’s chi-square test, the Fisher’s exact test, the Cochran-Armitage trend test, and odds ratios [4]. SNPs identified as significant by univariate analyses may be further examined for interactions among them using methods such as logistic regression.

### Genetic interactions

Genetic interactions, also known as epistasis, can be defined biologically as well as statistically. Biologically, epistasis refers to gene-gene interaction when the action of one gene is modified by one or several other genes. Statistically, epistasis refers to interaction between variants at multiple loci in which the total effect of the combination of variants at the different loci may differ considerably from a linear combination of the effects of individual loci. The detection of statistical epistasis has the potential to indicate genetic loci that have a biological interaction [5].

Statistical methods for identifying genetic interactions can be broadly divided into exhaustive and non-exhaustive methods. Exhaustive methods examine all possible SNP-subsets and examples include Multifactor Dimensionality Reduction (MDR) [6,7] and the BCM [3] that we describe in the next section. Examples of non-exhaustive methods include BOolean Operation-based Screening (BOOST), SNPHarvester, and SNPRuler. We briefly describe these methods below.

The software package PLINK that is used widely for the analysis of GWAS datasets implements logistic regression for the detection of SNP-SNP interactions in either all or specific sets of SNPs in a dataset [8].

MDR exhaustively evaluates all 1-, 2-, 3-, ..*n*-SNP subsets where *n* is specified by the user. It combines the variables in a SNP subset to construct a single binary variable and uses classification accuracy of the binary variable to evaluate a SNP-subset. Since MDR does not scale up beyond a few hundred SNPs, for high dimensional data a multivariate filtering algorithm called ReliefF is applied to reduce the number of SNPs to a few hundred [5,6,9,10].

BOOST uses a two-step procedure [11]. In the screening step, it uses an approximate likelihood ratio statistic that is computationally efficient and computes it for all pairs of SNPs. Only those SNPs that pass a threshold in the first step are examined for significant interaction effect using the classical likelihood ratio test that is computationally more expensive.

SNPHarvester is a stochastic search algorithm that uses a two-step procedure to identify epistatic interactions [12]. In the first step it identifies 40–50 significant SNP groups using a stochastic search strategy, and in the second step, it fits a penalized logistic regression model to each group.

SNPRuler searches in the space of SNP rules and uses a branch-and-bound strategy to prune the huge number of possible rules in GWAS data [13]. An example of a rule is *X*_1_ = 0 ^ *X*_2_ = 2 ⇒ *Z* = 1 (*X*_1_ and *X*_2_ are SNPs, the three genotypes that a SNP can take are coded as 0, 1 and 2 and *Z* is a binary outcome variable). The quality of a rule is evaluated with the chi-square statistic.

### Alzheimer’s disease

Alzheimer’s disease (AD) is the commonest neurodegenerative disease associated with aging and the commonest cause of dementia [14]. AD affects about 3% of all people between ages 65 and 74, about 19% of those between 75 and 84, and about 47% of those over 85. AD is characterized by adult onset of progressive dementia that typically begins with subtle memory failure and progresses to a slew of cognitive deficits like confusion, language disturbance and poor judgment [15].

AD is typically divided into early-onset Alzheimer’s disease (EOAD) in which the onset of disease is before 60 years of age and late-onset Alzheimer’s disease (LOAD) in which the onset is at or after 60 years of age. EOAD is rare and exhibits an autosomal dominant mode of inheritance. The genetic basis of EOAD is well established, and mutations in one of three genes (amyloid precursor protein gene - *APP*, presenilin 1 gene - *PSEN1*, or presenilin 2 gene - *PSEN2*) account for most cases of EOAD [16].

LOAD is widespread and is estimated to strike almost half of all people over the age of 85. LOAD is believed to be a disease with both genetic and environmental influences, and elucidating the role of genetic factors in the pathogenesis and development of LOAD has been a major focus of research for more than a decade. One genetic risk factor for LOAD that has been consistently replicated is the apolipoprotein E (*APOE*) locus determined by the combined genotypes at the loci rs429358 (APOE*4) and rs7412 (APOE*2) [17]. In the past few years, GWASs have identified several additional genetic loci associated with LOAD [18-22].

### Bayesian combinatorial method

BCM uses a Bayesian network (BN) to model a set of SNPs and interactions among them and their association with disease, and the model is evaluated with a Bayesian score. It then exhaustively searches a space of all possible models to identify high scoring models.

### Bayesian network model and score

**Figure 1:**
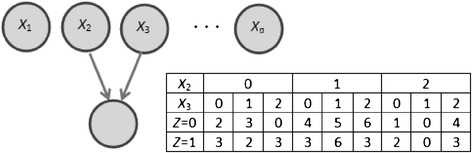
**A SNP Bayesian network model.** In this model SNPs *X*_2_ and *X*_3_ have an effect on *Z* and the remaining SNPs do not have an effect on *Z*. The table gives counts for the states of *Z* conditioned on the joint states of *X*_2_ and *X*_3_.

For a dataset *D* that contains a set of *n* SNPs {*X*_1_, *X*_2_, …, *X*_*n*_} and a binary outcome variable *Z* (e.g., disease or phenotype) on *N* individuals, BCM’s goal is to identify a set of SNPs that together are most predictive of *Z* in *D*. We model the effects of SNPs on *Z* with a BN that has *n* SNP-nodes and an additional node for *Z*. In this BN, which we call a SNP-BN, a subset of the *n* SNPs is modeled to have an effect on *Z* and every node in that subset has an arc to *Z* and every node not in the subset does not have an arc to *Z*. Also, there are no arcs between the SNP-nodes since we do not model the relations among the SNPs. Figure 1 gives an example of a SNP-BN where SNPs *X*_2_ and *X*_3_ are modeled to have a joint effect on *Z* (as shown by the arcs connecting them to *Z*) and the remaining SNPs do not have an effect on *Z*.

We evaluate the goodness of fit of a SNP-BN to data using an efficiently computable Bayesian score that computes the posterior probability of the BN given the data. In particular, we compute the BDeu (Bayesian Dirichlet equivalence uniform) score described in [23] which is commonly used in BN learning from data. This score is computed efficiently in closed form as follows:

1 $$ P\left(M\Big|D\right)=P(M)\prod_{i=1}^{n+1}\prod_{j=1}^{J_i}\frac{\Gamma \left({\alpha}_{ij}\right)}{\Gamma \left({n}_{ij}+{\alpha}_{ij}\right)}\prod_{k=1}^{K_i}\frac{\Gamma \left({n}_{ijk}+{\alpha}_{ijk}\right)}{\Gamma \left({\alpha}_{ijk}\right)} $$

where, Γ(⋅) is the gamma function, *M* is a SNP-BN, *P*(*D* | *M*) is the posterior probability of *M* given *D*, *P*(*M*) is the prior probability of *M*, *K*_*i*_ is the number of states of variable *X*_*i*_ represented by node *i*, *J*_*i*_ is the number of joint states of the parents of node *i*, *n*_*ijk*_ is the number of times in the data that node *i* is in state *k* given parent state *j*, *α*_*ijk*_ are the parameter priors in a Dirichlet distribution which define the prior probability over the BN parameters. Also, ${n}_{ij}={\displaystyle \sum_{k=1}^{K_i}{n}_{ijk},{\alpha}_{ij}}{\displaystyle \sum_{k=1}^{K_i}{\alpha}_{ijk}}$_,_ and ${\alpha}_{ijk}=\frac{\alpha }{J_i\cdot {K}_i}$, where *α* is a single user-defined parameter prior. The *n*_*ijk*_ are obtained from the data and stored in a counts table that is associated with each node (an example of a counts table for node *Z* is shown in Figure 1). We make the following assumptions and simplifications: (1) model the prior probability *P*(*M*) as a constant, i.e., *a priori* we consider all models to be equally plausible, (2) set *α =* 1 which is a commonly used non-informative parameter prior, (3) use the logarithmic form to simplify computations when dealing with very small numbers, and (4) assign the score for a SNP-BN model to be the BDeu score attributable to just node *Z* [24]. The reason for assumption (4) is as follows. The BDeu score decomposes over the nodes in the BN and each node makes an independent contribution to the overall score. In the space of SNP-BNs, the score contributions of the SNP-nodes is a constant since they have no incoming arcs, and hence variation in the scores for distinct SNP-BNs is due only to the score attributable to *Z*. Thus, the score we use for a SNP-BN is given by the following expression (index *i* is absent since there is only one node under consideration, namely, *Z*, and *K* = 2 since *Z* is binary):

2 $$ score(M)={\displaystyle \sum_{j=1}^J \log \frac{\Gamma \left({\alpha}_j\right)}{\Gamma \left({n}_j+{\alpha}_j\right)}+}{\displaystyle \sum_{k=1}^2 \log \frac{\Gamma \left({n}_{jk}+{\alpha}_{jk}\right)}{\Gamma \left({\alpha}_{jk}\right)}} $$

We have evaluated the BCM score in low dimensional synthetic data and found that in such data it has significantly greater power and is computed more efficiently than MDR [3,25,26].

In addition to computational efficiency and statistical power BCM has several additional advantages. The BCM score can be adapted to combine knowledge with data which has the potential to enhance the analysis of high dimensional GWAS datasets. Biological knowledge or results from analyses of earlier studies can be encoded in *P*(*M*) as a prior distribution over the models. The BN model used by BCM can be used for non-genetic variables such as environmental effects of disease. Thus, BCM can be used to identify combinations of genetic and environmental effects on disease. Finally, BCM can handle unbalanced datasets and its applicability is not restricted to datasets with approximately equal cases and controls.

## Materials & methods

This section describes the GWAS datasets, the experimental methods, and previously identified LOAD SNPs.

### GWAS dataset

We used two different LOAD GWAS datasets in our experiments. The first dataset was part of the University of Pittsburgh Alzheimer’s Disease Research Center (ADRC) that is described elsewhere [20]. This dataset consists of 2,245 individuals, of which 1290 had LOAD and 955 did not. For each individual, the genotype data consists of 682,685 SNPs on autosomal chromosomes.

The second dataset was collected by the Translational Genomics Research Institute (TGen) [22]. This dataset consists of 1,411 individuals, of which. 861 had LOAD and 550 did not. For each individual, the imputed genotype data consists of 234,665 SNPs on autosomal chromosomes. For each individual, the genotype data consists of 502,627 SNPs; the original investigators analyzed 312,316 SNPs after applying quality controls. We used those 312,316 SNPs, plus two additional *APOE* SNPs from the same study namely, rs429358 and rs7412.

The encoding of the genotypes followed the raw file format of the whole-genome analysis package PLINK, which contains a header line and then one line per individual. Each line has *v* + 6 fields (where *v* is the number of variants); the first six fields contain identification codes, the sex and the phenotype of the individual. The remaining fields contain variant genotypes, coded as a single allele dosage number (0, 1, 2 of minor allele) [8]. The University of Pittsburgh IRB approved the use of the datasets for the study.

### Experimental methods

BCM searches exhaustively over all possible SNP-BN models in a dataset. For a GWAS dataset with half a million SNPs, the number of SNP-BN models is 2^*n*^ = 9.95 × 10^150514^ and the number of SNP-BN models with just 2 SNPs is $$ \left(\begin{array}{c}\hfill 500000\hfill \\ {}\hfill 2\hfill \end{array}\right) $$ = 1.25 × 10^11^. Thus, the search space is very large and it is computationally infeasible to evaluate every model in the space [27].

We addressed this challenge by applying BCM to a restricted space of SNP-BN models that consisted of a subset of all possible 2-SNP models. We considered only those 2-SNP models where one of the SNPs in a model is a member of a set of SNPs previously known to be associated with LOAD and the second SNP is any SNP (excluding the first SNP) in the dataset of interest. Since the number of known LOAD associated SNPs is much smaller than the number of SNPs in a dataset, it was computationally tractable to search this space of SNP-BN models. The selection of the previously identified LOAD SNPs that we used is described in the next section.

We applied BCM to each of the two GWAS datasets separately and analyzed in detail the top scoring 200 SNP-BN models. We chose to examine the top 200 models because the model score decreased substantially after the 200 models. From each SNP-BN model, we extracted the SNP that was not in the set of previously identified LOAD SNPs. We mapped these SNPs to genes and considered only intragenic SNPs for further analyses. We performed the SNP to gene mapping with BioQ, a web-service which uses dbSNP build 135 and Genome Assembly GRCh37.p5 [28]. We performed enrichment analysis of the annotations of the associated genes in the Gene Ontology (GO) with the web-based tool GeneCoDis. For a set of genes GeneCoDis retrieves the associated GO terms, and identifies and ranks those GO terms that are significantly enriched in the set of genes [29,30]. Enriched functional descriptors facilitate the interpretation of the gene set. The hierarchical nature of the GO annotations however means that the set of enriched GO terms may contain terms closely related in a parent–child relationship [31]. Such redundant terms confound the interpretation. Therefore, we further examined the GO terms associated with the intragenic SNPs using the REViGo webserver. The REViGo software evaluates the semantic similarity between the enriched terms, identifies the most informative common ancestors and the related redundant GO terms and groups the latter under their ancestors [32]. The resulting set facilitates simultaneous examination of the enriched GO terms at two levels: a detailed one, at the lowest level overrepresented term and a more abstract one at the highest level common ancestor of overrepresented terms. The detailed level can reveal specific genes of interest whereas the abstract level serves a compact overview of the processes, functions and cellular compartments associated with the genes in the set.

In addition to the ontology analysis of the top scoring 200 SNP-BN models, we performed additional analyses of the top scoring 30 SNP-BN models. We analyzed the genes associated with the intragenic SNPs for differential expression in AD, through the ArrayExpress web server [33] and biological function analysis. Differential gene expression in relation to AD aims to integrate experimental evidence from transcriptomic analysis with those of genomic analysis. Up-regulation or down-regulation in AD of a gene in our results indicates increased biological plausibility for the reported genetic interaction. Finally, elements from the functional description of a gene (expression site, function related to the nervous system or pathways of LOAD, previous literature) were considered as supporting the biological relevance of an identified interaction.

We also compared BCM with logistic regression (LR) that is implemented in PLINK, since LR is typically used in genetic epidemiology and association studies. In PLINK, we used the --epistasis option that provides an LR test for interaction that assumes an allelic model [4,8]. We applied LR to the ADRC and the TGEN datasets to identify pairwise interactions between the dataset SNPs and the set of previously identified LOAD SNPs that was used with BCM.

### Previously identified LOAD SNPs

We obtained a set of SNPs that are known to be associated with LOAD from the AlzGene website. The AlzGene website contains a regularly updated database of SNPs that have been shown to be associated with LOAD mostly in GWAS studies [34]. The curators of the AlzGene website use criteria established by the Human Genome Epidemiology Network (HuGENet) for assessing the cumulative evidence of associations of SNPs with disease [35]. We obtained 10 SNPs that were assessed to have sufficiently strong evidence of being associated with LOAD from the AlzGene website in March 2012. If a previously identified LOAD SNP was not present in our datasets, we selected a replacement SNP. The replacement SNP was within 500 kb, in the same gene, as the original SNP with pairwise linkage disequilibrium threshold of r2 ≥ 0.8, using the SNAP web-based tool [36]. Using this protocol, we were unable to identify replacement SNPs in the TGen dataset for three previously identified LOAD SNPs; therefore we replaced them with SNPs from other genes, also reported as significantly associated with LOAD in the AlzGene website. Table 1 gives the list of 10 previously identified LOAD SNPs that we used in the experiments.

**Table 1:** **Previously identified LOAD SNPs**

**#**	**Gene**	**AlzGene SNP**	**Odds ratio (95% CI)**	**p value**	**ADRC SNP**	**r** ^**2**^	**TGen SNP**	**r** ^**2**^
1	APOE 4	rs429358	3.685 (3.30-4.12)	<1E-50	Same	-	Same	-
2	CR1	rs3818361	1.174 (1.14-1.21)	4.72E-21	Same	-	rs6656401	0.840
3	PICALM	rs3851179	0.879 (0.86-0.9)	2.85E-20	Same	-	rs7110631	0.841
4	MS4A6A	rs610932	0.904 (0.88-0.93)	1.81E-11	Same	-	rs574695	0.935
5	CD33	rs3865444	0.893 (0.86-0.93)	2.04E-10	Same	-	Same	-
6	MS4A4E	rs670139	1.079 (1.05-1.11)	9.51E-10	rs600550	1	rs676309	1
7	CD2AP	rs9349407	1.117 (1.08-1.16)	2.75E-09	rs9296559	1	rs9296558	1
8	GAB2	rs2373115	0.85 (0.76-0.94)		Same	-	Same	-
9	SORL1	rs2282649	1.10 (1.03-1.17)		rs726601	0.922	rs726601	0.922
10	TF	rs1049296	1.18 (1.06-1.31)		Same	-	Same	-

*AlzGene SNP*: the SNP in the AlzGene meta-analysis, along with the relevant odds ratios and p values (the latter for those SNPs with p values <0.00001); *ADRC SNP*: the corresponding SNP in the ADRC dataset, along with the r^2^ scores; *TGen SNP*: the corresponding SNP in the TGen dataset, along with the r^2^ scores for linkage disequilibrium.

## Results and discussion

This section describes the results that were obtained from applying BCM to the ADRC LOAD dataset and from applying BCM to the ADRC and the TGen GWAS datasets.

### Top scoring SNP-BN models

**Figure 2:**
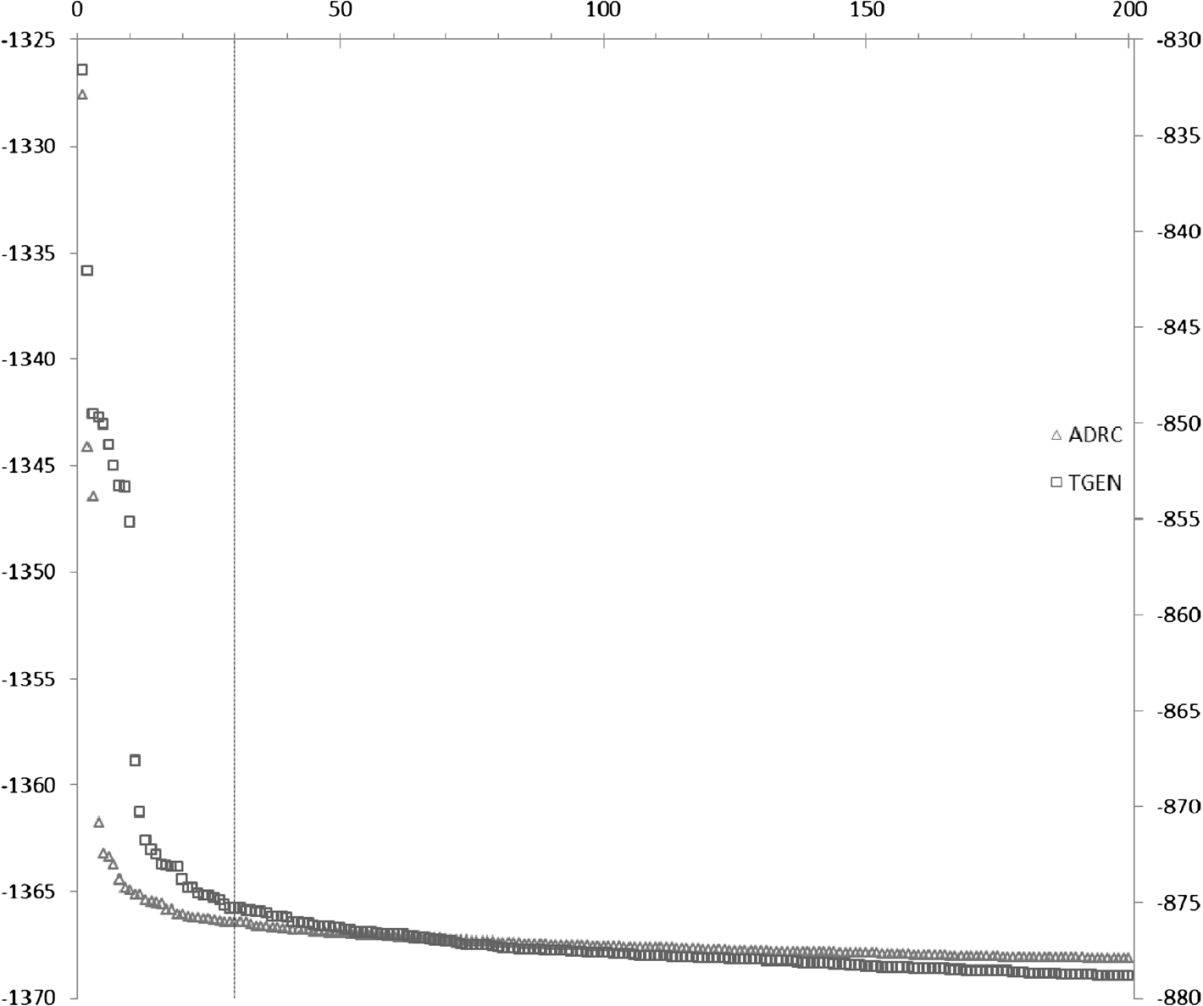
**Top 200 BCM model scores plot for both datasets tested.** Plots of the distribution of BCM model scores for the top ranked 200 SNP-BN models for the two datasets, ADRC and TGen. The scores for the ADRC dataset (blue points) correspond to the left hand Y axis, while those for the TGen dataset correspond to the right hand Y axis. The dotted vertical line marks the top ranked 200 SNP-BN models.

Each SNP-BN model includes two SNPs: one SNP is a previously identified LOAD SNP and the other is any SNP from the dataset. We call the former SNP a *known SNP* and the latter SNP a *dataset SNP*. The known SNP and the dataset SNP from the top scoring 200 SNP-BN models are given in Additional file 1: Table S1 (for ADRC) and Additional file 1: Table S2 (for TGen) in the Supplemental Tables. A plot of the scores of the top scoring 200 SNP-BN models for the two datasets is shown in Figure 2.

In the ADRC dataset, the known SNP in each of the top scoring 200 SNP-BN models is rs429358 (APOE*4). The dataset SNPs from the top scoring 200 models included 92 intragenic SNPs mapping to 77 genes.

In the TGen dataset, the known SNP in SNP-BN models ranked 1 and 10 to 200 is rs429358 and the dataset SNPs included 82 intragenic SNPs that mapped to 69 genes. In the 8 SNP-BN models ranked 2–9 the known SNP was located on genes *GAB2*, *MS4A6A*, *MS4A4E*, *CR1*, *PICALM*, *SORL1*, *TF* and the dataset SNP for all 8 models is rs7412. SNPs rs429358 and rs7412 are located on the *APOE* gene and their combined genotypes determine the APOE allelic status which is known to be the strongest genetic variant that is predictive of LOAD.

A comparison of the top scoring 200 SNP-BN models for the ADRC and TGEN datasets shows that they have in common two dataset SNPs, rs7412 (*APOE* gene) and rs4420638 (*APOC1* gene). Additionally, two of the intragenic dataset SNPs from each dataset map to genes *CAMK1D* (rs11257738 in ADRC and rs17151584 in TGen) and *FBXL13* (rs7779121 in ADRC and rs17475512 in TGen).

### GO term analysis

The most informative common ancestors of the overrepresented GO terms obtained from GeneCoDis for the ADRC dataset are given in Additional file 1: Table S3 and for the TGen dataset are given in Additional file 1: Table S4 in the Supplemental Tables. In both sets nervous system-related terms are enriched (e.g., *regulation of dendrite development, nervous system development, regulation of axon extension, short term memory*), as well as terms related to cholesterol and lipid metabolism (e.g., *lipid metabolic process*, *chylomicron*), beta amyloid (*beta amyloid binding*) cell membranes (e.g., *integral to membrane, plasma membrane*, *postsynaptic, clathrin-coated endocytic vesicle*), calmodulin and intracellular calcium homeostasis (e.g., *calmodulin binding, cytosolic calcium ion transport*) and the immune system (*immunoglobulin binding*). Overrepresentation of these terms shows that the identified genes from both datasets include genes that are members of biochemical pathways involved in LOAD pathophysiology [17,37].

### Expression analysis

The 15 genes corresponding to the 18 dataset intragenic SNPs from the top scoring 30 models in the ADRC dataset and the 19 genes corresponding to the 19 dataset intragenic SNPs from the top scoring 30 models in the TGen dataset were examined for relative expression in AD (see Table 2 for the ADRC dataset and and see Table 3 for the TGen dataset). In these tables, the second to last column gives the rank of the corresponding SNP based on the model score obtained by applying BCM to 1-SNP models. For some of the SNPs the rank based on the 1-SNP model is very low compared to the score of the corresponding 2-SNP model which implies that these SNPS would not have been identified by univariate analysis. The last column gives the p values for the pairs of SNPs that were obtained from using logistic regression in PLINK.

**Table 2:** **Functional description and expression of genes associated with the top 30 dataset SNPs in the ADRC dataset**

**Gene symbol** **(SNP)**	**Name**	**Description**	**Expression in AD**	**1-SNP model rank**	**p value of pair from PLINK**
***APOC1*** (rs4420638)	Apolipoprotein C-I	Appears to modulate the interaction of APOE with beta-migrating VLDL. Binds free fatty acids.	Overexpressed [38]	2	0.3326
***TOMM40*** (rs157582)	Translocase of outer mitochondrial membrane 40 homolog	Channel-forming subunit of the translocase of the mitochondrial outer membrane (TOM) complex, essential for protein import into mitochondria.	Underexpressed [38]	3	0.4139
***APOE*** (rs7412)	Apolipoprotein E	APOE is essential for the normal catabolism of triglyceride-rich lipoprotein constituents. Known risk factor for LOAD.	Overexpressed [38]	5	0.8172
***SNTG1*** (rs16914489)	Gamma-1-syntrophin	Specifically expressed in the brain, highly expressed in the cortex. Organizes the subcellular localization of a variety of proteins.	Overexpressed [38]	24906	0.004546
***TMEM217*** (rs9470543)	Transmembrane protein 217	Expressed in the brain	-	4584	0.004643
***SMAD6*** (rs3934907)	Mothers against DPP homolog 6	Negative regulation of BMP and TGF-beta/activin-signaling. BMP-6 is increased in AD brains and leads to impaired neurogenesis [39]. Reduced TGF-beta signaling is involved in neurodegeneration and promotes AD like changes in mice [40].	Underexpressed [38]	41282	0.0000998
***NPAS3*** (rs4981180)	Neuronal PAS domain protein 3.	Transcription factor. May regulate genes involved in neurogenesis. Associated with schizophrenia and mental retardation	Overexpressed [38]	1086	0.1225
***NTM*** (rs11222692)	Neurotrimin	May promote neurite outgrowth and adhesion. NTM lies at locus 11q25, which has been associated with AD [41,42].	Overexpressed [38]	12209	0.1422
***PPAPDC1A*** (rs4752432)	Phosphatidic acid phosphatase type 2 domain containing 1A	-	-	6852	0.3963
***NPFF*** (rs8192593)	Neuropeptide FF-amide peptide precursor	Modulation of morphine-induced antinociception.	-	3981	0.1251
***SLC25A21*** (rs7140725)	Solute carrier family 25	Known also as ornithine decarboxylase (ODC). Mitochondrial oxoadipate carrier, part of polyamine synthesis pathway.	Overexpressed [43,44]	444	0.06767
***RAB23*** (rs182662)	Member RAS oncogene family	Intracellular protein transportation. Regulated by miRNA155, which also regulates PICALM (a known AD association).	Underexpressed [38]	96	0.08251
***UNC5D*** (rs4577954)	unc-5 homolog D (C. elegans)	Netrin receptor: netrins are secreted proteins that direct axon extension and cell migration during neural development. APP also binds Netrin-1 and in transgenic mice this suppresses amyloid beta peptide production [45].	-	63972	0.6731
***CHD9*** (rs3852742)	Chromodomain helicase DNA binding protein 9, PPARA -interacting complex 320 kDa protein	Transcriptional co-activator for PPARA. The APOE gene promoter has a binding site for PPAR alpha. Low CHD9 activity could reduce APOE levels. Increase in APOE transcription has been shown to clear amyloid beta in AD mouse models [46].	Overexpressed [38]	1061	0.04696
***CNTN4*** (rs9819935)	Contactin 4, Brain-derived immunoglobulin superfamily protein 2	Mainly expressed in brain. Neuronal membrane protein that may play a role in the formation of axon connections in the developing nervous system. Associated with Spinocerebellar Ataxia, Amyotrophic Lateral Sclerosis, 3p deletion syndrome.	-	2149	0.002386

*1-SNP model rank:* rank of the corresponding SNP in terms of univariate 1-SNP model score.

**Table 3:** **Functional description and expression of genes associated with the top 30 dataset SNPs in the TGen dataset**

**Gene symbol (SNP)**	**Name**	**Description**	**Differential expression in AD**	**1-SNP model rank**	**p value of pair from PLINK**
***APOE2*** (rs7412)	Apolipoprotein E	APOE is essential for the normal catabolism of triglyceride-rich lipoprotein constituents. Known risk factor for LOAD.	Overexpressed [38]	1	0.05993
***APOC1*** (rs4420638)	Apolipoprotein C-1	Appears to modulate the interaction of APOE with beta-migrating VLDL. Binds free fatty acids.	Overexpressed [38]	3	0.705
***C10orf11*** (rs7079348)	Chromosome 10 open reading frame 11	A brain-expressed gene. Haploinsufficiency of C10orf11 contributes to the cognitive defects in 10q22 syndrome [47].	-	4	0.009623
***VWC2*** (rs10499687)	von Willebrand factor C domain-containing protein 2 (Brorin, Brain-specific chordin-like protein)	Encodes a secreted bone morphogenic protein (BMP) antagonist. The encoded protein is possibly involved in neural function and development and may have a role in cell adhesion. BMP-6 is increased in AD brains and leads to impaired neurogenesis [39].	Underexpressed [48]	12	0.7698
***PSD3*** (rs17126808)	Pleckstrin and Sec7 domain containing 3	Guanine nucleotide exchange factor for ARF6 that contributes to the regulation of dendritic branching [49].	Overexpressed [38]	34	0.001623
***GXYLT2*** (rs3732443)	Glucoside xylosyltransferase 2	Elongates the O-linked glucose attached to EGF-like repeats in the extracellular domain of Notch proteins [49], which are substrates of γ-secretase, the enzyme involved in amyloid beta production [50].	Underexpressed in a murine AD model [51]	6	0.211
***GABBR2*** (rs2779550)	Gamma-aminobutyric acid (GABA) B receptor, 2	Target for autophagy regulation in neurodegenerative diseases [52].	Overexpressed [38]	391	0.0002945
***ENPP2*** (rs16892852)	Ectonucleotide pyrophosphatase/phosphodiesterase 2	Hydrolyzes lysophospholipids to produce lysophosphatidic acid (LPA) in extracellular fluids. Predominantly expressed in brain, placenta, ovary, and small intestine. Secreted by most body fluids including serum and cerebrospinal fluid [49].	Overexpressed [38]	92	0.04851
***GLP1R*** (rs910171)	Glucagon-like peptide 1 receptor	Member of the glucagon receptor family (also includes glucagon, GLP-2, secretin, GHRH and GIP receptors).In the brain located in hypothalamus and brainstem. Protective against amyloid beta accumulation in rats [53].	Overexpressed [38]	193	0.01462
***MOSC1*** (rs746767)	MOCO sulphurase C-terminal domain containing 1	A mitochondrial oxidoreductase, cofactor: molybdenum, is expressed in the brain. MOSC1 is a target for miR-129-5p, like GABBR2, and miR-155, like PICALM.	-	66	0.04507
***TM4SF20*** (rs4408717)	Transmembrane 4 L six family member 20	Tetraspannin superfamily member. Tetraspanins are often thought to act as scaffolding proteins, anchoring multiple proteins to one area of the cell membrane. Other tetraspanin superfamily members have been implicated in Notch signaling and g-secretase activity modulation [54].	-	95	0.004495

*1-SNP model rank:* rank of the corresponding SNP in terms of univariate 1-SNP model score.

### Comparison of BCM with logistic regression applied to the ADRC and TGEN datasets

In the ADRC dataset, comparing the 200 top-ranking SNP-BN models of BCM with the top 200 SNP pairs of LR we have 5 models in common. In the case of the TGEN dataset there are 9 models in common between the 200 top-ranking SNP-BN models of BCM and the top 200 SNP pairs of LR. A comparison of the top 200 SNP pairs obtained with LR from the ADRC and TGEN datasets reveals no SNP pair in common.

### Discussion

Examining all pairs of SNPs in a GWAS dataset for identifying interacting SNP pairs is usually not computationally tractable due to the large number of SNP pairs. We addressed this challenge by examining only a subset of all pairs of SNPs where one member of the pair is drawn from a small set of previously known disease-associated SNPs and by using a Bayesian score to evaluate that statistical association of a SNP pair with the disease. We applied this strategy to two LOAD GWAS datasets and our results show that it can identify interacting SNPs of plausible biological significance. Moreover, this strategy finds SNPs that would be overlooked in a univariate analysis because they exhibit small main effects; however, they are detected when paired with another SNP due to interaction effects.

Evaluation of the identified SNPs did not include experimental validation in the laboratory. Instead, we gathered evidence from the literature that supports that the SNPs we identified may play a role in the biological mechanisms underlying LOAD. To do so, we selected the intragenic SNPs and identified the relevant genes. We used ontology enrichment analysis of the list of genes to provide an overview of the functions of the identified genes. We then used descriptions in gene databases to examine each gene for appropriate context (tissue expression, function, product protein interactions). Finally, we examined the literature for studies reporting association of the gene, of the gene transcript (expression studies) or the encoded protein with LOAD.

In both LOAD GWAS datasets that we examined, the previously known disease-associated SNP that was identified is either rs429358 (APOE*4) or rs7412 (APOE*2); these SNPs reside in the *APOE* gene which is known to be the strongest genetic determinant for LOAD. GO term enrichment analysis of the dataset SNPs identified terms that are relevant to biochemical pathways implicated in the pathogenesis of LOAD such as *lipid metabolic process*, *calmodulin binding, nervous system development* and multiple membrane-related terms.

Gene expression analysis of the dataset SNPs showed that for each dataset studied a majority of the genes corresponding to the top 30 dataset SNPs are differentially expressed in LOAD. Functional annotations and literature evidence that are presented with the expression data in the relevant tables further support the role of these genes in the pathogenesis of LOAD.

Among the genes corresponding to the top 200 dataset SNPs, besides the *APOE* gene, three other genes are common to both datasets: *APOC1*, *CAMK1D* and *FBXL13*. Evidence supporting the interaction of *APOC1* with *APOE* is presented in the analysis for the top 30 dataset SNPs. The second gene in common is the *CAMK1D* (calcium/calmodulin-dependent protein kinase ID) gene that encodes a member of the Ca2+/calmodulin-dependent protein kinase 1 subfamily of serine/threonine kinases family of calmodulin kinases [55], which modulate neuronal development and plasticity [56]. The gene is overexpressed in AD and is expressed in the brain especially during hippocampal formation with high expression in the pyramidal cell layers [38,57]. *CAMK1D* interacts physically with *CALM1* (calmodulin), which has been associated with AD risk [58]. The CAMK1D protein may regulate calcium-mediated granulocyte function and activates MAPK3 (Mitogen-activated protein kinase 3). It phosphorylates -in vitro- the transcription factor CREM (cAMP responsive element binding) isoform beta and probably CREB1 [57]. The CREB pathway is involved in memory formation and CREB phosphorylation has been proposed as a signalling pathway involved in the pathogenesis of AD [59], while CREB pathway down-regulation may have a role in exacerbations of AD [57]. Another member of the serine/threonine kinases family of calmodulin kinases, the neuronal CaM kinase II phosphorylates tau protein on ser262, an important step in the formation of neurofibrillary tangles in AD [60]. The third gene in common between the two datasets is the *FBXL13* (F-box and leucine-rich repeat protein 13) gene that encodes a protein belonging to the F-box protein family. Members of this family have a characteristic approximately 40-amino acid F-box motif and take part in SCF (SKP1-CUL1-F-box protein) complexes that act as protein-ubiquitin ligases [55]. The ubiquitin-proteasome system is involved in protein turnover and degradation and is perturbed in AD [61]. An SCF complex of another F box protein (FBXW7) is probably involved in the degradation of PSEN1 protein [49].

In addition to the genes corresponding to the top 30 top scoring SNP-BN models in the ADRC dataset, we found other genes in lower scoring SNP-BN models with plausible associations with LOAD. In the 80^th^ scoring model (dataset SNP rs7793977), gene *PION* [pigeon homolog (Drosophila)], also known as *GSAP* (gamma-secretase-activating protein), is known to increase amyloid beta production [55]. In the 196^th^ scoring model, (dataset SNP rs6534145), the *PDE5A* (phosphodiesterase 5A, cGMP-specific) gene could be implicated to LOAD pathogenesis via two different mechanisms. PDE5A is a substrate of CASP3 (caspase 3) [62], which in turn has been shown to be involved in the early synaptic dysfunction in a mouse model of AD [51]. It has also been shown that inhibition of PDE5A results in a decrease in the transcription of Wnt/β-catenin [63]. A reduction in Wnt signalling has been implicated in the amyloid beta-dependent neurodegeneration in LOAD [64].

While BCM has been applied to low dimensional synthetic data with good results [24], in this paper we have applied it to GWAS datasets. BCM has several advantages. It is computationally more efficient than the widely used MDR [3]. Since BCM uses the Bayesian paradigm, the BCM score represents a coherent way to combine knowledge with data. Biological knowledge or results from analyses of earlier studies can be encoded as a prior distribution over the models that can then be used in Equation 1. Use of informative priors is becoming common in the analysis of microarray expression studies, and a similar strategy can be employed for genomic data.

In this paper we applied our method to two GWAS datasets, as replication is considered to be a necessary step in the validation of GWAS findings [65]. However, many markers identified through GWAS have failed to replicate. Proposed explanations for this include environmental interactions, genetic heterogeneity, inadequate statistical power and population differences, i.e. inter-population differences in the minor allele frequencies (MAF). It has been shown that racial differences directly influence the odds ratios of validated markers, an explanation being the differing MAF among populations [66]. In addition, results of simulation studies have shown that even small differences in allelic frequencies can affect the detection of main as well as of interaction effect, and may lead to non-replication or even reversal of the direction of the association [67]. Therefore, failure to replicate a finding across two different populations should not necessarily be viewed as proof of lack of true association of the initial finding.

A limitation of our study is the use of GWAS datasets related to a single disease, although it is an important disease. In future research, we plan to apply and investigate the utility of BCM on GWAS datasets related to additional diseases. Another limitation is the use just 10 previously known LOAD-associated SNPs. In future work, we plan to explore the use of a larger set of known LOAD associated SNPs that will include SNPs with weaker evidence of being associated with LOAD. In addition, we plan to study the effect of excluding the *APOE* SNPs rs429358 and rs7412 which are present in every SNP pair we examined for biological plausibility. Another limitation is that we did not use informative prior probabilities for encoding prior knowledge from the literature and previous GWASs. BCM can be extended easily to allow the incorporation of informative priors and inclusion of informative priors in the analysis is an interesting area for study.

## Conclusion

We applied BCM to two LOAD GWAS datasets to identify pairs of SNPs that in combination have high statistical association with development of LOAD. To reduce the large search space of all possible parts of SNPs in a GWAS dataset we restricted BCM to evaluate those SNP pairs where one of the SNP was drawn from a set of 10 previously known LOAD associated SNPs. Our results identified several SNPs that have biological evidence of being involved in the pathogenesis of LOAD that would not have been identified by univariate analysis alone due to small main effect but were identified in conjunction with another SNP. These results provide support for applying BCM to identify potential genetic variants such as SNPs from high dimensional GWAs datasets.

## Additional file

Additional file 1:
**Top scoring 200 SNP-BN models in the ADRC dataset (Table S1) and the TGen dataset (Table S2) and significantly overrepresented GO terms related to the genes in the top 200 dataset SNPs in the ADRC dataset (Table S3) and in the TGen dataset (Table S4).**

## References

[1] Hardy J, Singleton A (2009). Genomewide association studies and human disease. N Engl J Med.

[2] Thornton-Wells TA, Moore JH, Haines JL (2004). Genetics, statistics and human disease: analytical retooling for complexity. Trends Genet.

[3] Visweswaran S, Wong A-KI, Barmada MM (2009). A Bayesian method for identifying genetic interactions. AMIA Annu Symp Proc.

[4] Balding DJ (2006). A tutorial on statistical methods for population association studies. Nat Rev Genet.

[5] Cordell HJ (2009). Detecting gene-gene interactions that underlie human diseases. Nat Rev Genet.

[6] Hahn LW, Ritchie MD, Moore JH (2003). Multifactor dimensionality reduction software for detecting gene-gene and gene-environment interactions. Bioinformatics.

[7] Moore JH, Gilbert JC, Tsai C-T, Chiang F-T, Holden T, Barney N, White BC (2006). A flexible computational framework for detecting, characterizing, and interpreting statistical patterns of epistasis in genetic studies of human disease susceptibility. J Theor Biol.

[8] Purcell S, Neale B, Todd-Brown K, Thomas L, Ferreira MAR, Bender D, Maller J, Sklar P, de Bakker PIW, Daly MJ, Sham PC (2007). PLINK: a tool Set for whole-genome association and population-based linkage analyses. Am J Hum Genet.

[9] Ritchie MD, Hahn LW, Roodi N, Bailey LR, Dupont WD, Parl FF, Moore JH (2001). Multifactor-dimensionality reduction reveals high-order interactions among estrogen-metabolism genes in sporadic breast cancer. Am J Hum Genet.

[10] Moore J, White B: **Tuning ReliefF for genome-wide genetic analysis.** *Evol Comput Mach Learn Data Min Bioinformatics* 2007, **4447**:166–175.

[11] Wan X, Yang C, Yang Q, Xue H, Fan X, Tang NLS, Yu W (2010). BOOST: A fast approach to detecting gene-gene interactions in genome-wide case–control studies. Am J Hum Genet.

[12] Yang C, He Z, Wan X, Yang Q, Xue H, Yu W (2009). SNPHarvester: a filtering-based approach for detecting epistatic interactions in genome-wide association studies. Bioinformatics.

[13] Wan X, Yang C, Yang Q, Xue H, Tang NLS, Yu W (2010). Predictive rule inference for epistatic interaction detection in genome-wide association studies. Bioinformatics.

[14] Goedert M, Spillantini MG (2006). A century of Alzheimer’s disease. Science.

[15] Bertram L, Lill CM, Tanzi RE (2010). The genetics of Alzheimer disease: back to the future. Neuron.

[16] Avramopoulos D (2009). Genetics of Alzheimer’s disease: recent advances. Genome Med.

[17] Holtzman DM, Morris JC, Goate AM (2011). Alzheimer’s disease: the challenge of the second century. Sci Transl Med.

[18] Wijsman EM, Pankratz ND, Choi Y, Rothstein JH, Faber KM, Cheng R, Lee JH, Bird TD, Bennett DA, Diaz-Arrastia R, Goate AM, Farlow M, Ghetti B, Sweet RA, Foroud TM, Mayeux R (2011). Genome-wide association of familial late-onset Alzheimer’s disease replicates BIN1 and CLU and nominates CUGBP2 in interaction with APOE. PLoS Genet.

[19] Hu X, Pickering E, Liu YC, Hall S, Fournier H, Katz E, Dechairo B, John S, Van Eerdewegh P, Soares H (2011). Meta-analysis for genome-wide association study identifies multiple variants at the BIN1 locus associated with late-onset Alzheimer’s disease. PLoS One.

[20] Kamboh MI, Demirci FY, Wang X, Minster RL, Carrasquillo MM, Pankratz VS, Younkin SG, Saykin AJ, Jun G, Baldwin C, Logue MW, Buros J, Farrer L, Pericak-Vance MA, Haines JL, Sweet RA, Ganguli M, Feingold E, DeKosky ST, Lopez OL, Barmada MM (2012). Genome-wide association study of Alzheimer’s disease. Transl Psychiatry.

[21] Hollingworth P, Harold D, Sims R, Gerrish A, Lambert J-C, Carrasquillo MM, Abraham R, Hamshere ML, Pahwa JS, Moskvina V, Dowzell K, Jones N, Stretton A, Thomas C, Richards A, Ivanov D, Widdowson C, Chapman J, Lovestone S, Powell J, Proitsi P, Lupton MK, Brayne C, Rubinsztein DC, Gill M, Lawlor B, Lynch A, Brown KS, Passmore PA, Craig D (2011). Common variants at ABCA7, MS4A6A/MS4A4E, EPHA1, CD33 and CD2AP are associated with Alzheimer’s disease. Nat Genet.

[22] Reiman EM, Webster JA, Myers AJ, Hardy J, Dunckley T, Zismann VL, Joshipura KD, Pearson JV, Hu-Lince D, Huentelman MJ, Craig DW, Coon KD, Liang WS, Herbert RH, Beach T, Rohrer KC, Zhao AS, Leung D, Bryden L, Marlowe L, Kaleem M, Mastroeni D, Grover A, Heward CB, Ravid R, Rogers J, Hutton ML, Melquist S, Petersen RC, Alexander GE (2007). GAB2 alleles modify Alzheimer’s risk in APOE epsilon4 carriers. Neuron.

[23] Heckerman D, Geiger D, Chickering DM: **Learning Bayesian Networks: The Combination of Knowledge and Statistical Data.** *Mach Learn* 1995, **20:**197–243.

[24] Visweswaran S, Wong A-KI (2009). Bayesian combinatorial partitioning for detecting interactions among genetic variants. Summit Transl Bioinformatics.

[25] Jiang X, Neapolitan RE, Barmada MM, Visweswaran S (2011). Learning genetic epistasis using Bayesian network scoring criteria. BMC Bioinformatics.

[26] Jiang X, Barmada MM, Visweswaran S (2010). Identifying genetic interactions in genome-wide data using Bayesian networks. Genet Epidemiol.

[27] Ritchie MD (2011). Using biological knowledge to uncover the mystery in the search for epistasis in genome-wide association studies. Ann Hum Genet.

[28] Saccone SF, Quan J, Jones PL (2012). BioQ: tracing experimental origins in public genomic databases using a novel data provenance model. Bioinformatics.

[29] Carmona-Saez P, Chagoyen M, Tirado F, Carazo JM, Pascual-Montano A (2007). GENECODIS: a web-based tool for finding significant concurrent annotations in gene lists. Genome Biol.

[30] Nogales-Cadenas R, Carmona-Saez P, Vazquez M, Vicente C, Yang X, Tirado F, Carazo JM, Pascual-Montano A (2009). GeneCodis: interpreting gene lists through enrichment analysis and integration of diverse biological information. Nucleic Acids Res.

[31] Khatri P, Drăghici S (2005). Ontological analysis of gene expression data: current tools, limitations, and open problems. Bioinformatics.

[32] Supek F, Bošnjak M, Škunca N, Šmuc T (2011). REVIGO summarizes and visualizes long lists of gene ontology terms. PLoS One.

[33] Parkinson H, Sarkans U, Kolesnikov N, Abeygunawardena N, Burdett T, Dylag M, Emam I, Farne A, Hastings E, Holloway E, Kurbatova N, Lukk M, Malone J, Mani R, Pilicheva E, Rustici G, Sharma A, Williams E, Adamusiak T, Brandizi M, Sklyar N, Brazma A (2011). ArrayExpress update–an archive of microarray and high-throughput sequencing-based functional genomics experiments. Nucleic Acids Res.

[34] Bertram L, McQueen MB, Mullin K, Blacker D, Tanzi RE (2007). Systematic meta-analyses of Alzheimer disease genetic association studies: the AlzGene database. Nat Genet.

[35] Ioannidis JPA, Boffetta P, Little J, O’Brien TR, Uitterlinden AG, Vineis P, Balding DJ, Chokkalingam A, Dolan SM, Flanders WD, Higgins JPT, McCarthy MI, McDermott DH, Page GP, Rebbeck TR, Seminara D, Khoury MJ (2008). Assessment of cumulative evidence on genetic associations: interim guidelines. Int J Epidemiol.

[36] Johnson AD, Handsaker RE, Pulit SL, Nizzari MM, O’Donnell CJ, de Bakker PIW (2008). SNAP: a web-based tool for identification and annotation of proxy SNPs using HapMap. Bioinformatics.

[37] Morgan K (2011). The three new pathways leading to Alzheimer’s disease. Neuropathol Appl Neurobiol.

[38] Lukk M, Kapushesky M, Nikkilä J, Parkinson H, Goncalves A, Huber W, Ukkonen E, Brazma A (2010). A global map of human gene expression. Nat Biotechnol.

[39] Crews L, Adame A, Patrick C, Delaney A, Pham E, Rockenstein E, Hansen L, Masliah E (2010). Increased BMP6 levels in the brains of Alzheimer’s disease patients and APP transgenic mice are accompanied by impaired neurogenesis. J Neurosci.

[40] Tesseur I, Zou K, Esposito L, Bard F, Berber E, Can JV, Lin AH, Crews L, Tremblay P, Mathews P, Mucke L, Masliah E, Wyss-Coray T (2006). Deficiency in neuronal TGF-beta signaling promotes neurodegeneration and Alzheimer’s pathology. J Clin Invest.

[41] Liu F, Arias-Vásquez A, Sleegers K, Aulchenko YS, Kayser M, Sanchez-Juan P, Feng B-J, Bertoli-Avella AM, van Swieten J, Axenovich TI, Heutink P, van Broeckhoven C, Oostra BA, van Duijn CM (2007). A genomewide screen for late-onset Alzheimer disease in a genetically isolated Dutch population. Am J Hum Genet.

[42] Blacker D, Bertram L, Saunders AJ, Moscarillo TJ, Albert MS, Wiener H, Perry RT, Collins JS, Harrell LE, Go RCP, Mahoney A, Beaty T, Fallin MD, Avramopoulos D, Chase GA, Folstein MF, McInnis MG, Bassett SS, Doheny KJ, Pugh EW, Tanzi RE (2003). Results of a high-resolution genome screen of 437 Alzheimer’s disease families. Hum Mol Genet.

[43] Nilsson T, Bogdanovic N, Volkman I, Winblad B, Folkesson R, Benedikz E (2006). Altered subcellular localization of ornithine decarboxylase in Alzheimer’s disease brain. Biochem Biophys Res Commun.

[44] Bernstein HG, Müller M (1999). The cellular localization of the L-ornithine decarboxylase/polyamine system in normal and diseased central nervous systems. Prog Neurobiol.

[45] Lourenço FC, Galvan V, Fombonne J, Corset V, Llambi F, Müller U, Bredesen DE, Mehlen P (2009). Netrin-1 interacts with amyloid precursor protein and regulates amyloid-beta production. Cell Death Differ.

[46] Cramer PE, Cirrito JR, Wesson DW, Lee CYD, Karlo JC, Zinn AE, Casali BT, Restivo JL, Goebel WD, James MJ, Brunden KR, Wilson DA, Landreth GE (2012). ApoE-directed therapeutics rapidly clear β-amyloid and reverse deficits in AD mouse models. Science.

[47] Tzschach A, Bisgaard A-M, Kirchhoff M, Graul-Neumann LM, Neitzel H, Page S, Ahmed A, Müller I, Erdogan F, Ropers H-H, Kalscheuer VM, Ullmann R (2010). Chromosome aberrations involving 10q22: report of three overlapping interstitial deletions and a balanced translocation disrupting C10orf11. Eur J Hum Genet.

[48] Webster JA, Gibbs JR, Clarke J, Ray M, Zhang W, Holmans P, Rohrer K, Zhao A, Marlowe L, Kaleem M, McCorquodale DS, Cuello C, Leung D, Bryden L, Nath P, Zismann VL, Joshipura K, Huentelman MJ, Hu-Lince D, Coon KD, Craig DW, Pearson JV, Heward CB, Reiman EM, Stephan D, Hardy J, Myers AJ (2009). Genetic control of human brain transcript expression in Alzheimer disease. Am J Hum Genet.

[49] Consortium TU (2012). Reorganizing the protein space at the Universal Protein Resource (UniProt). Nucleic Acids Res.

[50] Frykman S, Teranishi Y, Hur J-Y, Sandebring A, Goto Yamamoto N, Ancarcrona M, Nishimura T, Winblad B, Bogdanovic N, Schedin-Weiss S, Kihara T, Tjernberg LO (2012). Identification of two novel synaptic γ-secretase associated proteins that affect amyloid β-peptide levels without altering Notch processing. Neurochem Int.

[51] D’Amelio M, Cavallucci V, Middei S, Marchetti C, Pacioni S, Ferri A, Diamantini A, De Zio D, Carrara P, Battistini L, Moreno S, Bacci A, Ammassari-Teule M, Marie H, Cecconi F (2011). Caspase-3 triggers early synaptic dysfunction in a mouse model of Alzheimer’s disease. Nat Neurosci.

[52] Lipinski MM, Zheng B, Lu T, Yan Z, Py BF, Ng A, Xavier RJ, Li C, Yankner BA, Scherzer CR, Yuan J (2010). Genome-wide analysis reveals mechanisms modulating autophagy in normal brain aging and in Alzheimer’s disease. Proc Natl Acad Sci U S A.

[53] Perry T, Greig NH (2005). Enhancing central nervous system endogenous GLP-1 receptor pathways for intervention in Alzheimer’s disease. Curr Alzheimer Res.

[54] Dunn CD, Sulis ML, Ferrando AA, Greenwald I (2010). A conserved tetraspanin subfamily promotes Notch signaling in Caenorhabditis elegans and in human cells. Proc Natl Acad Sci U S A.

[55] Maglott D, Ostell J, Pruitt KD, Tatusova T (2011). Entrez Gene: gene-centered information at NCBI. Nucleic Acids Res.

[56] Wayman GA, Lee Y-S, Tokumitsu H, Silva AJ, Soderling TR (2008). Calmodulin-kinases: modulators of neuronal development and plasticity. Neuron.

[57] Pugazhenthi S, Wang M, Pham S, Sze C-I, Eckman CB (2011). Downregulation of CREB expression in Alzheimer’s brain and in Aβ-treated rat hippocampal neurons. Mol Neurodegener.

[58] Lambert J-C, Grenier-Boley B, Chouraki V, Heath S, Zelenika D, Fievet N, Hannequin D, Pasquier F, Hanon O, Brice A, Epelbaum J, Berr C, Dartigues J-F, Tzourio C, Campion D, Lathrop M, Amouyel P (2010). Implication of the immune system in Alzheimer’s disease: evidence from genome-wide pathway analysis. J Alzheimers Dis.

[59] Müller M, Cárdenas C, Mei L, Cheung K-H, Foskett JK (2011). Constitutive cAMP response element binding protein (CREB) activation by Alzheimer’s disease presenilin-driven inositol trisphosphate receptor (InsP3R) Ca2+ signaling. Proc Natl Acad Sci U S A.

[60] Yamauchi T (2005). Neuronal Ca2+/calmodulin-dependent protein kinase II–discovery, progress in a quarter of a century, and perspective: implication for learning and memory. Biol Pharm Bull.

[61] Riederer BM, Leuba G, Vernay A, Riederer IM (2011). The role of the ubiquitin proteasome system in Alzheimer’s disease. Exp Biol Med (Maywood).

[62] Frame M, Wan KF, Tate R, Vandenabeele P, Pyne NJ (2001). The gamma subunit of the rod photoreceptor cGMP phosphodiesterase can modulate the proteolysis of two cGMP binding cGMP-specific phosphodiesterases (PDE6 and PDE5) by caspase-3. Cell Signal.

[63] Tinsley HN, Gary BD, Keeton AB, Lu W, Li Y, Piazza GA (2011). Inhibition of PDE5 by sulindac sulfide selectively induces apoptosis and attenuates oncogenic Wnt/β-catenin-mediated transcription in human breast tumor cells. Cancer Prev Res (Phila).

[64] Inestrosa NC, Toledo EM (2008). The role of Wnt signaling in neuronal dysfunction in Alzheimer’s Disease. Mol Neurodegener.

[65] Chanock SJ, Manolio T, Boehnke M, Boerwinkle E, Hunter DJ, Thomas G, Hirschhorn JN, Abecasis G, Altshuler D, Bailey-Wilson JE, Brooks LD, Cardon LR, Daly M, Donnelly P, Fraumeni JF, Freimer NB, Gerhard DS, Gunter C, Guttmacher AE, Guyer MS, Harris EL, Hoh J, Hoover R, Kong CA, Merikangas KR, Morton CC, Palmer LJ, Phimister EG, Rice JP, NCI-NHGRI Working Group on Replication in Association Studies (2007). Replicating genotype-phenotype associations. Nature.

[66] Ioannidis JPA (2007). Non-replication and inconsistency in the genome-wide association setting. Hum Hered.

[67] Greene CS, Penrod NM, Williams SM, Moore JH (2009). Failure to replicate a genetic association may provide important clues about genetic architecture. PLoS One.

