# Supplementary material for: Identifying genetic interactions associated with late-onset Alzheimer’s disease
Source: BioData Min. 2014 Dec 19;7:35. doi: 10.1186/s13040-014-0035-z (PMC4300162; doi:10.1186/s13040-014-0035-z)
Supplement: Additional file 1: — Top scoring 200 SNP-BN models in the ADRC dataset (Table S1) and the TGen dataset (Table S2) and significantly overrepresented GO terms related to the genes in the top 200 dataset SNPs in the ADRC dataset (Table S3) and in the TGen dataset (Table S4). [file 13040_2014_35_MOESM1_ESM.doc]

**Additional file 1: Table S1**. Top scoring 200 SNP-BN models in the ADRC dataset. (MA: minor allele, Chr: chromosome number)

| Rank | Known SNP | MA | Dataset SNP | MA | Chr. |
| --- | --- | --- | --- | --- | --- |
| 1 | rs429358 | G | rs4420638 | G | 19 |
| 2 | rs429358 | G | rs157582 | T | 19 |
| 3 | rs429358 | G | rs2075650 | G | 19 |
| 4 | rs429358 | G | rs1555110 | T | 6 |
| 5 | rs429358 | G | rs7412 | A | 19 |
| 6 | rs429358 | G | rs16914489 | A | 8 |
| 7 | rs429358 | G | rs12632905 | A | 3 |
| 8 | rs429358 | G | rs4761019 | C | 12 |
| 9 | rs429358 | G | rs9470543 | A | 6 |
| 10 | rs429358 | G | rs16979372 | G | 19 |
| 11 | rs429358 | G | rs6840147 | A | 4 |
| 12 | rs429358 | G | rs2001696 | A | 14 |
| 13 | rs429358 | G | rs3934907 | A | 15 |
| 14 | rs429358 | G | rs4981180 | T | 14 |
| 15 | rs429358 | G | rs11222692 | T | 11 |
| 16 | rs429358 | G | rs4752432 | T | 10 |
| 17 | rs429358 | G | rs8192593 | A | 12 |
| 18 | rs429358 | G | rs246994 | A | 5 |
| 19 | rs429358 | G | rs6507833 | C | 18 |
| 20 | rs429358 | G | rs16950202 | T | 15 |
| 21 | rs429358 | G | rs992558 | A | 5 |
| 22 | rs429358 | G | rs7140725 | C | 14 |
| 23 | rs429358 | G | rs182662 | T | 6 |
| 24 | rs429358 | G | rs4577954 | G | 8 |
| 25 | rs429358 | G | rs3852742 | A | 16 |
| 26 | rs429358 | G | rs9819935 | C | 3 |
| 27 | rs429358 | G | rs6843926 | C | 4 |
| 28 | rs429358 | G | rs4361760 | T | 8 |
| 29 | rs429358 | G | rs6549883 | G | 3 |
| 30 | rs429358 | G | rs6004582 | A | 22 |
| 31 | rs429358 | G | rs10513305 | G | 3 |
| 32 | rs429358 | G | rs1040425 | A | 1 |
| 33 | rs429358 | G | rs207363 | C | 4 |
| 34 | rs429358 | G | rs2973910 | A | 5 |
| 35 | rs429358 | G | rs13254708 | T | 8 |
| 36 | rs429358 | G | rs7769321 | G | 6 |
| 37 | rs429358 | G | rs8125585 | T | 20 |
| 38 | rs429358 | G | rs1835655 | A | 18 |
| 39 | rs429358 | G | rs1325550 | C | 6 |
| 40 | rs429358 | G | rs7668712 | A | 4 |
| 41 | rs429358 | G | rs9895280 | G | 17 |
| 42 | rs429358 | G | rs16916040 | T | 9 |
| 43 | rs429358 | G | rs11697010 | G | 20 |
| 44 | rs429358 | G | rs7309534 | A | 12 |
| 45 | rs429358 | G | rs1314016 | G | 9 |
| 46 | rs429358 | G | rs6777958 | G | 3 |
| 47 | rs429358 | G | rs16908426 | T | 8 |
| 48 | rs429358 | G | rs12580173 | T | 12 |
| 49 | rs429358 | G | rs17027130 | C | 2 |
| 50 | rs429358 | G | rs2817158 | A | 1 |
| 51 | rs429358 | G | rs6755466 | A | 2 |
| 52 | rs429358 | G | rs7766672 | G | 6 |
| 53 | rs429358 | G | rs16908400 | A | 8 |
| 54 | rs429358 | G | rs10810649 | A | 9 |
| 55 | rs429358 | G | rs7598244 | C | 2 |
| 56 | rs429358 | G | rs10862591 | G | 12 |
| 57 | rs429358 | G | rs17066794 | T | 8 |
| 58 | rs429358 | G | rs12462846 | G | 19 |
| 59 | rs429358 | G | rs12541339 | G | 8 |
| 60 | rs429358 | G | rs16908405 | G | 8 |
| 61 | rs429358 | G | rs741553 | T | 3 |
| 62 | rs429358 | G | rs16916098 | C | 9 |
| 63 | rs429358 | G | rs1778540 | T | 1 |
| 64 | rs429358 | G | rs12873187 | A | 13 |
| 65 | rs429358 | G | rs16875750 | A | 8 |
| 66 | rs429358 | G | rs2724856 | G | 2 |
| 67 | rs429358 | G | rs9811266 | G | 3 |
| 68 | rs429358 | G | rs204190 | G | 9 |
| 69 | rs429358 | G | rs7563563 | G | 2 |
| 70 | rs429358 | G | rs16897993 | G | 4 |
| 71 | rs429358 | G | rs16916079 | T | 9 |
| 72 | rs429358 | G | rs10884225 | A | 10 |
| 73 | rs429358 | G | rs9473701 | G | 6 |
| 74 | rs429358 | G | rs12073615 | G | 1 |
| 75 | rs429358 | G | rs3864087 | A | 3 |
| 76 | rs429358 | G | rs1809401 | T | 4 |
| 77 | rs429358 | G | rs3845668 | A | 2 |
| 78 | rs429358 | G | rs17145881 | A | 5 |
| 79 | rs429358 | G | rs10230004 | A | 7 |
| 80 | rs429358 | G | rs7793977 | G | 7 |
| 81 | rs429358 | G | rs10467182 | C | 12 |
| 82 | rs429358 | G | rs1778541 | C | 1 |
| 83 | rs429358 | G | rs11257738 | A | 10 |
| 84 | rs429358 | G | rs7075330 | C | 10 |
| 85 | rs429358 | G | rs1869866 | A | 3 |
| 86 | rs429358 | G | rs8044050 | C | 16 |
| 87 | rs429358 | G | rs1551680 | T | 10 |
| 88 | rs429358 | G | rs2786498 | A | 1 |
| 89 | rs429358 | G | rs17022467 | T | 2 |
| 90 | rs429358 | G | rs12889998 | T | 14 |
| 91 | rs429358 | G | rs2154153 | C | 9 |
| 92 | rs429358 | G | rs11951324 | G | 5 |
| 93 | rs429358 | G | rs12249088 | A | 10 |
| 94 | rs429358 | G | rs2223 | T | 14 |
| 95 | rs429358 | G | rs1182534 | A | 20 |
| 96 | rs429358 | G | rs17025271 | T | 2 |
| 97 | rs429358 | G | rs12435937 | C | 14 |
| 98 | rs429358 | G | rs193096 | T | 19 |
| 99 | rs429358 | G | rs13135278 | A | 4 |
| 100 | rs429358 | G | rs10015280 | C | 4 |
| 101 | rs429358 | G | rs7913357 | A | 10 |
| 102 | rs429358 | G | rs9565267 | T | 13 |
| 103 | rs429358 | G | rs6919481 | A | 6 |
| 104 | rs429358 | G | rs7749306 | T | 6 |
| 105 | rs429358 | G | rs16935300 | G | 10 |
| 106 | rs429358 | G | rs2236902 | G | 1 |
| 107 | rs429358 | G | rs10836114 | A | 11 |
| 108 | rs429358 | G | rs17024694 | G | 12 |
| 109 | rs429358 | G | rs11976749 | T | 7 |
| 110 | rs429358 | G | rs12604943 | C | 18 |
| 111 | rs429358 | G | rs16952149 | A | 18 |
| 112 | rs429358 | G | rs16955202 | A | 16 |
| 113 | rs429358 | G | rs1454540 | C | 2 |
| 114 | rs429358 | G | rs11180676 | C | 12 |
| 115 | rs429358 | G | rs2039611 | A | 9 |
| 116 | rs429358 | G | rs16884109 | G | 8 |
| 117 | rs429358 | G | rs10046684 | G | 8 |
| 118 | rs429358 | G | rs9469637 | A | 6 |
| 119 | rs429358 | G | rs10932849 | G | 2 |
| 120 | rs429358 | G | rs7314773 | A | 12 |
| 121 | rs429358 | G | rs7013633 | C | 8 |
| 122 | rs429358 | G | rs4792630 | T | 17 |
| 123 | rs429358 | G | rs11111323 | A | 12 |
| 124 | rs429358 | G | rs11901178 | A | 2 |
| 125 | rs429358 | G | rs10142229 | T | 14 |
| 126 | rs429358 | G | rs6927181 | C | 6 |
| 127 | rs429358 | G | rs12586381 | C | 14 |
| 128 | rs429358 | G | rs10827343 | T | 10 |
| 129 | rs429358 | G | rs17751194 | A | 11 |
| 130 | rs429358 | G | rs12366983 | A | 12 |
| 131 | rs429358 | G | rs7779121 | C | 7 |
| 132 | rs429358 | G | rs12110995 | T | 6 |
| 133 | rs429358 | G | rs7642557 | C | 3 |
| 134 | rs429358 | G | rs17736782 | A | 6 |
| 135 | rs429358 | G | rs3123247 | C | 10 |
| 136 | rs429358 | G | rs12870493 | C | 13 |
| 137 | rs429358 | G | rs7572966 | T | 2 |
| 138 | rs429358 | G | rs11620945 | A | 14 |
| 139 | rs429358 | G | rs3736456 | C | 4 |
| 140 | rs429358 | G | rs17095485 | T | 14 |
| 141 | rs429358 | G | rs2582860 | T | 10 |
| 142 | rs429358 | G | rs17071079 | C | 3 |
| 143 | rs429358 | G | rs405509 | G | 19 |
| 144 | rs429358 | G | rs17224968 | C | 8 |
| 145 | rs429358 | G | rs10266589 | C | 7 |
| 146 | rs429358 | G | rs755836 | T | 12 |
| 147 | rs429358 | G | rs6732115 | C | 2 |
| 148 | rs429358 | G | rs1003580 | A | 22 |
| 149 | rs429358 | G | rs17016018 | G | 4 |
| 150 | rs429358 | G | rs10846183 | G | 12 |
| 151 | rs429358 | G | rs280632 | A | 2 |
| 152 | rs429358 | G | rs17027638 | G | 3 |
| 153 | rs429358 | G | rs2272866 | T | 1 |
| 154 | rs429358 | G | rs7008028 | G | 8 |
| 155 | rs429358 | G | rs12063989 | C | 1 |
| 156 | rs429358 | G | rs7621621 | A | 3 |
| 157 | rs429358 | G | rs3788374 | C | 22 |
| 158 | rs429358 | G | rs11608431 | A | 12 |
| 159 | rs429358 | G | rs16872402 | G | 7 |
| 160 | rs429358 | G | rs741232 | T | 19 |
| 161 | rs429358 | G | rs2929103 | T | 8 |
| 162 | rs429358 | G | rs751247 | C | 14 |
| 163 | rs429358 | G | rs12006360 | T | 9 |
| 164 | rs429358 | G | rs578991 | G | 19 |
| 165 | rs429358 | G | rs10125000 | T | 9 |
| 166 | rs429358 | G | rs12203621 | A | 6 |
| 167 | rs429358 | G | rs3018066 | G | 4 |
| 168 | rs429358 | G | rs9497952 | A | 6 |
| 169 | rs429358 | G | rs7950864 | T | 11 |
| 170 | rs429358 | G | rs881795 | T | 6 |
| 171 | rs429358 | G | rs7759004 | C | 6 |
| 172 | rs429358 | G | rs504350 | A | 19 |
| 173 | rs429358 | G | rs11257374 | C | 10 |
| 174 | rs429358 | G | rs2151182 | C | 9 |
| 175 | rs429358 | G | rs1159993 | G | 12 |
| 176 | rs429358 | G | rs283129 | C | 5 |
| 177 | rs429358 | G | rs2038865 | T | 6 |
| 178 | rs429358 | G | rs6071006 | A | 20 |
| 179 | rs429358 | G | rs11876903 | G | 18 |
| 180 | rs429358 | G | rs10497718 | A | 2 |
| 181 | rs429358 | G | rs4402798 | G | 2 |
| 182 | rs429358 | G | rs2246646 | A | 18 |
| 183 | rs429358 | G | rs10072098 | C | 5 |
| 184 | rs429358 | G | rs16890450 | C | 6 |
| 185 | rs429358 | G | rs10157386 | C | 1 |
| 186 | rs429358 | G | rs1456922 | A | 3 |
| 187 | rs429358 | G | rs10167154 | C | 2 |
| 188 | rs429358 | G | rs7702926 | G | 5 |
| 189 | rs429358 | G | rs16853027 | A | 2 |
| 190 | rs429358 | G | rs497321 | A | 15 |
| 191 | rs429358 | G | rs7788611 | C | 7 |
| 192 | rs429358 | G | rs17015078 | G | 1 |
| 193 | rs429358 | G | rs4521036 | A | 2 |
| 194 | rs429358 | G | rs4800618 | C | 18 |
| 195 | rs429358 | G | rs17067325 | A | 13 |
| 196 | rs429358 | G | rs6534145 | A | 4 |
| 197 | rs429358 | G | rs2224215 | C | 1 |
| 198 | rs429358 | G | rs12550518 | C | 8 |
| 199 | rs429358 | G | rs11608933 | C | 12 |
| 200 | rs429358 | G | rs2405046 | T | 3 |

**Table S2.** Top scoring 200 SNP-BN models in the TGen dataset. (MA: minor allele, Chr: chromosome number)

| Rank | Known SNP | MA | Dataset SNP | MA | Chr. |
| --- | --- | --- | --- | --- | --- |
| 1 | rs429358 | T | rs7412 | T | 19 |
| 2 | rs2373115 | A | rs7412 | T | 19 |
| 3 | rs574695 | C | rs7412 | T | 19 |
| 4 | rs676309 | C | rs7412 | T | 19 |
| 5 | rs6656401 | T | rs7412 | T | 19 |
| 6 | rs7110631 | C | rs7412 | T | 19 |
| 7 | rs726601 | T | rs7412 | T | 19 |
| 8 | rs1049296 | T | rs7412 | T | 19 |
| 9 | rs9296558 | T | rs7412 | T | 19 |
| 10 | rs429358 | T | rs4420638 | G | 19 |
| 11 | rs429358 | T | rs7079348 | T | 10 |
| 12 | rs429358 | T | rs16967491 | T | 15 |
| 13 | rs429358 | T | rs6717497 | G | 2 |
| 14 | rs429358 | T | rs1950636 | A | 14 |
| 15 | rs429358 | T | rs10499687 | T | 7 |
| 16 | rs429358 | T | rs2122339 | A | 4 |
| 17 | rs429358 | T | rs3760114 | C | 16 |
| 18 | rs429358 | T | rs17126808 | G | 8 |
| 19 | rs429358 | T | rs2517509 | T | 6 |
| 20 | rs429358 | T | rs3732443 | G | 3 |
| 21 | rs429358 | T | rs2779550 | T | 9 |
| 22 | rs429358 | T | rs12671823 | T | 7 |
| 23 | rs429358 | T | rs383407 | G | 10 |
| 24 | rs429358 | T | rs16892852 | C | 8 |
| 25 | rs429358 | T | rs17013939 | A | 2 |
| 26 | rs429358 | T | rs910171 | C | 6 |
| 27 | rs429358 | T | rs4300052 | G | 9 |
| 28 | rs429358 | T | rs746767 | G | 1 |
| 29 | rs429358 | T | rs4408717 | G | 2 |
| 30 | rs429358 | T | rs2739771 | T | 15 |
| 31 | rs429358 | T | rs11170573 | A | 12 |
| 32 | rs429358 | T | rs6018172 | C | 20 |
| 33 | rs429358 | T | rs934745 | G | 18 |
| 34 | rs429358 | T | rs17756423 | G | 2 |
| 35 | rs429358 | T | rs7817227 | T | 8 |
| 36 | rs2373115 | A | rs4420638 | G | 19 |
| 37 | rs429358 | T | rs10508364 | G | 10 |
| 38 | rs429358 | T | rs11985044 | G | 8 |
| 39 | rs429358 | T | rs13044152 | A | 20 |
| 40 | rs429358 | T | rs17280356 | C | 5 |
| 41 | rs429358 | T | rs1210715 | T | 22 |
| 42 | rs429358 | T | rs16846058 | G | 1 |
| 43 | rs429358 | T | rs41479848 | G | 14 |
| 44 | rs429358 | T | rs1396702 | T | 16 |
| 45 | rs429358 | T | rs6486444 | G | 12 |
| 46 | rs429358 | T | rs903639 | T | 4 |
| 47 | rs429358 | T | rs4362167 | G | 11 |
| 48 | rs429358 | T | rs41412544 | G | 1 |
| 49 | rs429358 | T | rs13333829 | C | 16 |
| 50 | rs429358 | T | rs16909497 | T | 10 |
| 51 | rs429358 | T | rs17064302 | T | 8 |
| 52 | rs429358 | T | rs6508182 | C | 18 |
| 53 | rs429358 | T | rs6593919 | A | 1 |
| 54 | rs429358 | T | rs4356530 | A | 17 |
| 55 | rs429358 | T | rs16938663 | T | 8 |
| 56 | rs429358 | T | rs816470 | A | 5 |
| 57 | rs429358 | T | rs12535674 | C | 7 |
| 58 | rs429358 | T | rs7236694 | T | 18 |
| 59 | rs429358 | T | rs4394475 | C | 9 |
| 60 | rs429358 | T | rs17209127 | A | 11 |
| 61 | rs429358 | T | rs2140641 | A | 7 |
| 62 | rs429358 | T | rs17048904 | A | 4 |
| 63 | rs429358 | T | rs16903767 | T | 5 |
| 64 | rs429358 | T | rs6583904 | A | 10 |
| 65 | rs429358 | T | rs16974268 | C | 15 |
| 66 | rs429358 | T | rs17330779 | A | 7 |
| 67 | rs429358 | T | rs230305 | T | 1 |
| 68 | rs429358 | T | rs7198681 | C | 16 |
| 69 | rs429358 | T | rs7585710 | C | 2 |
| 70 | rs429358 | T | rs17151584 | G | 10 |
| 71 | rs429358 | T | rs7097398 | T | 10 |
| 72 | rs429358 | T | rs7979117 | A | 12 |
| 73 | rs429358 | T | rs1553903 | G | 4 |
| 74 | rs429358 | T | rs4771333 | C | 13 |
| 75 | rs429358 | T | rs188429 | T | 9 |
| 76 | rs429358 | T | rs9879473 | T | 3 |
| 77 | rs429358 | T | rs11842721 | A | 13 |
| 78 | rs429358 | T | rs12942139 | T | 17 |
| 79 | rs429358 | T | rs6094514 | T | 20 |
| 80 | rs429358 | T | rs7277699 | A | 21 |
| 81 | rs429358 | T | rs2522 | G | 10 |
| 82 | rs429358 | T | rs9562355 | A | 13 |
| 83 | rs429358 | T | rs17831322 | A | 8 |
| 84 | rs429358 | T | rs9982394 | T | 21 |
| 85 | rs429358 | T | rs10905444 | G | 10 |
| 86 | rs429358 | T | rs10115381 | C | 9 |
| 87 | rs429358 | T | rs12658567 | G | 5 |
| 88 | rs429358 | T | rs10517069 | C | 4 |
| 89 | rs429358 | T | rs11096511 | T | 2 |
| 90 | rs429358 | T | rs16967411 | G | 13 |
| 91 | rs429358 | T | rs17407781 | G | 10 |
| 92 | rs429358 | T | rs12673707 | A | 7 |
| 93 | rs429358 | T | rs17710010 | G | 14 |
| 94 | rs429358 | T | rs2184380 | T | 10 |
| 95 | rs429358 | T | rs11625439 | A | 14 |
| 96 | rs429358 | T | rs475093 | G | 1 |
| 97 | rs429358 | T | rs6972352 | T | 7 |
| 98 | rs429358 | T | rs7001472 | A | 8 |
| 99 | rs429358 | T | rs2374656 | G | 12 |
| 100 | rs429358 | T | rs573399 | A | 18 |
| 101 | rs429358 | T | rs16967391 | T | 13 |
| 102 | rs429358 | T | rs41461248 | A | 4 |
| 103 | rs429358 | T | rs17475512 | G | 7 |
| 104 | rs429358 | T | rs868535 | A | 10 |
| 105 | rs429358 | T | rs8119865 | C | 20 |
| 106 | rs429358 | T | rs16942981 | C | 18 |
| 107 | rs429358 | T | rs1983539 | G | 3 |
| 108 | rs429358 | T | rs2428192 | A | 6 |
| 109 | rs429358 | T | rs2913719 | C | 5 |
| 110 | rs429358 | T | rs3007246 | T | 13 |
| 111 | rs429358 | T | rs17139138 | A | 7 |
| 112 | rs429358 | T | rs4379923 | C | 13 |
| 113 | rs429358 | T | rs10824310 | T | 10 |
| 114 | rs429358 | T | rs17226741 | C | 16 |
| 115 | rs429358 | T | rs17473848 | C | 8 |
| 116 | rs429358 | T | rs11600112 | C | 11 |
| 117 | rs429358 | T | rs582790 | G | 11 |
| 118 | rs429358 | T | rs623758 | A | 9 |
| 119 | rs429358 | T | rs2333612 | C | 14 |
| 120 | rs429358 | T | rs9838256 | G | 3 |
| 121 | rs429358 | T | rs2225741 | T | 9 |
| 122 | rs429358 | T | rs4658692 | C | 1 |
| 123 | rs429358 | T | rs1671413 | T | 8 |
| 124 | rs429358 | T | rs12050816 | C | 15 |
| 125 | rs429358 | T | rs1275996 | G | 2 |
| 126 | rs429358 | T | rs10272660 | G | 7 |
| 127 | rs429358 | T | rs17048190 | G | 2 |
| 128 | rs429358 | T | rs4565533 | G | 9 |
| 129 | rs429358 | T | rs11694103 | A | 2 |
| 130 | rs429358 | T | rs6035760 | C | 20 |
| 131 | rs429358 | T | rs10976715 | G | 9 |
| 132 | rs429358 | T | rs313142 | G | 4 |
| 133 | rs429358 | T | rs6453333 | G | 5 |
| 134 | rs429358 | T | rs1022055 | G | 5 |
| 135 | rs429358 | T | rs3118548 | A | 9 |
| 136 | rs429358 | T | rs10406148 | A | 19 |
| 137 | rs429358 | T | rs6550942 | T | 3 |
| 138 | rs429358 | T | rs4669836 | A | 2 |
| 139 | rs429358 | T | rs7077757 | T | 10 |
| 140 | rs429358 | T | rs10905447 | C | 10 |
| 141 | rs429358 | T | rs11118165 | A | 1 |
| 142 | rs429358 | T | rs7299181 | T | 12 |
| 143 | rs429358 | T | rs215924 | T | 6 |
| 144 | rs429358 | T | rs17279525 | G | 1 |
| 145 | rs429358 | T | rs4780141 | T | 15 |
| 146 | rs429358 | T | rs6747871 | G | 2 |
| 147 | rs429358 | T | rs1477599 | C | 15 |
| 148 | rs429358 | T | rs1345186 | T | 3 |
| 149 | rs429358 | T | rs752123 | A | 2 |
| 150 | rs429358 | T | rs1038891 | A | 11 |
| 151 | rs429358 | T | rs1021135 | G | 3 |
| 152 | rs429358 | T | rs16847008 | G | 2 |
| 153 | rs429358 | T | rs10823349 | A | 10 |
| 154 | rs429358 | T | rs17170955 | T | 7 |
| 155 | rs429358 | T | rs17119283 | A | 11 |
| 156 | rs429358 | T | rs16941373 | T | 17 |
| 157 | rs429358 | T | rs17041561 | C | 2 |
| 158 | rs429358 | T | rs2242452 | G | 10 |
| 159 | rs429358 | T | rs41495846 | C | 19 |
| 160 | rs429358 | T | rs641205 | A | 18 |
| 161 | rs429358 | T | rs6536071 | C | 4 |
| 162 | rs429358 | T | rs16940197 | T | 15 |
| 163 | rs429358 | T | rs17151710 | T | 5 |
| 164 | rs429358 | T | rs17216650 | G | 3 |
| 165 | rs429358 | T | rs17104648 | G | 10 |
| 166 | rs429358 | T | rs543352 | A | 13 |
| 167 | rs429358 | T | rs4749805 | T | 10 |
| 168 | rs429358 | T | rs2779556 | A | 9 |
| 169 | rs429358 | T | rs10466295 | C | 10 |
| 170 | rs429358 | T | rs10513196 | A | 3 |
| 171 | rs429358 | T | rs1325874 | T | 10 |
| 172 | rs429358 | T | rs877196 | C | 12 |
| 173 | rs429358 | T | rs4964503 | C | 12 |
| 174 | rs429358 | T | rs2615994 | T | 1 |
| 175 | rs429358 | T | rs11038071 | A | 11 |
| 176 | rs429358 | T | rs4852482 | A | 2 |
| 177 | rs429358 | T | rs16912248 | C | 9 |
| 178 | rs429358 | T | rs223226 | C | 1 |
| 179 | rs429358 | T | rs4404123 | A | 17 |
| 180 | rs429358 | T | rs17452064 | C | 10 |
| 181 | rs429358 | T | rs5999604 | A | 22 |
| 182 | rs429358 | T | rs11900757 | G | 2 |
| 183 | rs429358 | T | rs10509602 | C | 10 |
| 184 | rs429358 | T | rs7232965 | G | 18 |
| 185 | rs429358 | T | rs16936670 | C | 8 |
| 186 | rs429358 | T | rs7904521 | A | 10 |
| 187 | rs429358 | T | rs8110293 | G | 19 |
| 188 | rs429358 | T | rs16856031 | A | 2 |
| 189 | rs429358 | T | rs12430052 | G | 13 |
| 190 | rs429358 | T | rs4749807 | G | 10 |
| 191 | rs429358 | T | rs17115455 | A | 1 |
| 192 | rs429358 | T | rs16905332 | A | 8 |
| 193 | rs429358 | T | rs10510121 | T | 10 |
| 194 | rs429358 | T | rs11832371 | A | 12 |
| 195 | rs429358 | T | rs7838203 | G | 8 |
| 196 | rs429358 | T | rs17160942 | T | 7 |
| 197 | rs429358 | T | rs7582851 | A | 2 |
| 198 | rs429358 | T | rs4666189 | T | 2 |
| 199 | rs429358 | T | rs4778564 | C | 15 |
| 200 | rs429358 | T | rs6972615 | G | 7 |

**Table S3.** Significantly overrepresented GO terms related to the genes in the top 200 dataset SNPs in ADRC dataset.

|  | **GO term identifier** | **GO term description** | **Gene Symbol** |
| --- | --- | --- | --- |
| **BP** | GO:0033700 | phospholipid efflux | APOC1, APOE |
|  | *GO:0033344* | *cholesterol efflux* | *APOC1, APOE* |
|  | *GO:0032368* | *regulation of lipid transport* | *APOC1* |
|  | *GO:0032374* | *regulation of cholesterol transport* | *APOC1* |
|  | *GO:0032375* | *negative regulation of cholesterol transport* | *APOC1* |
|  | GO:0060282 | positive regulation of oocyte development | PDE5A, PDE3A |
|  | *GO:0002021* | *response to dietary excess* | *APOE* |
|  | GO:0031589 | cell-substrate adhesion | SMAD6, VWF |
|  | *GO:0007155* | *cell adhesion* | *ADAM12, VWF, CNTN4, NTM, PDZD2* |
|  | GO:0032060 | bleb assembly | MYLK |
|  | GO:0090162 | establishment of epithelial cell polarity | PARD3 |
|  | GO:0008356 | asymmetric cell division | PARD3 |
|  | GO:0046135 | pyrimidine nucleoside catabolic process | NT5E, DPYD |
|  | *GO:0009166* | *nucleotide catabolic process* | *NT5E* |
|  | *GO:0009187* | *cyclic nucleotide metabolic process* | *PDE5A* |
|  | *GO:0006214* | *thymidine catabolic process* | *DPYD* |
|  | *GO:0006222* | *UMP biosynthetic process* | *DPYD* |
|  | GO:0007154 | cell communication | SLC8A1, SNTG1 |
|  | GO:0042157 | lipoprotein metabolic process | APOC1, APOE |
|  | GO:0006629 | lipid metabolic process | APOC1, GDPD4, APOE, PDE3A |
|  | GO:0034587 | piRNA metabolic process | TDRD9 |
|  | GO:0090073 | positive regulation of protein homodimerization activity | CRBN |
|  | GO:0060401 | cytosolic calcium ion transport | SLC8A1 |
|  | GO:0019934 | cGMP-mediated signaling | APOE, PDE3A |
|  | *GO:0030828* | *positive regulation of cGMP biosynthetic process* | *APOE* |
|  | *GO:0007191* | *activation of adenylate cyclase activity by dopamine receptor signaling pathway* | *GNAL, OPRM1* |
|  | *GO:0007193* | *inhibition of adenylate cyclase activity by G-protein signaling pathway* | *GNAL, OPRM1* |
|  | GO:0071918 | urea transmembrane transport | SLC14A2 |
|  | *GO:0015840* | *urea transport* | *SLC14A2* |
|  | GO:0043457 | regulation of cellular respiration | PRDM16 |
|  | GO:0006641 | triglyceride metabolic process | APOC1, APOE |
|  | GO:0006003 | fructose 2,6-bisphosphate metabolic process | PFKFB3 |
|  | GO:0034616 | response to laminar fluid shear stress | SMAD6 |
|  | GO:0071321 | cellular response to cGMP | PDE3A |
|  | GO:0030512 | negative regulation of transforming growth factor beta receptor signaling pathway | SMAD6, PRDM16 |
|  | *GO:0010991* | *negative regulation of SMAD protein complex assembly* | *SMAD6* |
|  | *GO:0060394* | *negative regulation of pathway-restricted SMAD protein phosphorylation* | *SMAD6* |
|  | GO:0006554 | lysine catabolic process | SLC25A21 |
|  | GO:0055086 | nucleobase-containing small molecule metabolic process | NT5E, DPYD |
|  | GO:0042159 | lipoprotein catabolic process | APOE |
|  | *GO:0042158* | *lipoprotein biosynthetic process* | *APOE* |
|  | GO:0006196 | AMP catabolic process | NT5E |
|  | *GO:0046069* | *cGMP catabolic process* | *PDE5A* |
|  | GO:0010793 | regulation of mRNA export from nucleus | ZC3H3 |
|  | *GO:0016973* | *poly(A)+ mRNA export from nucleus* | *ZC3H3* |
|  | GO:0032488 | Cdc42 protein signal transduction | APOE |
|  | GO:0007352 | zygotic specification of dorsal/ventral axis | SMAD6 |
|  | *GO:0021513* | *spinal cord dorsal/ventral patterning* | *RAB23* |
|  | GO:0034447 | very-low-density lipoprotein particle clearance | APOC1, APOE |
|  | *GO:0034375* | *high-density lipoprotein particle remodeling* | *APOC1,APOE* |
|  | *GO:0034380* | *high-density lipoprotein particle assembly* | *APOE* |
|  | *GO:0034379* | *very-low-density lipoprotein particle assembly* | *APOC1* |
|  | *GO:0034384* | *high-density lipoprotein particle clearance* | *APOE* |
|  | *GO:0034382* | *chylomicron remnant clearance* | *APOC1, APOE* |
|  | *GO:0034372* | *very-low-density lipoprotein particle remodeling* | *APOE* |
|  | *GO:0034369* | *plasma lipoprotein particle remodeling* | *APOC1* |
|  | *GO:0010916* | *negative regulation of very-low-density lipoprotein particle clearance* | *APOC1* |
|  | GO:0006145 | purine base catabolic process | DPYD |
|  | *GO:0006212* | *uracil catabolic process* | *DPYD* |
|  | *GO:0006210* | *thymine catabolic process* | *DPYD* |
|  | *GO:0006208* | *pyrimidine base catabolic process* | *DPYD* |
|  | *GO:0006207* | *'de novo' pyrimidine base biosynthetic process* | *DPYD* |
|  | *GO:0006206* | *pyrimidine base metabolic process* | *NT5E, DPYD* |
|  | GO:0009414 | response to water deprivation | SLC14A2 |
|  | *GO:0071476* | *cellular hypotonic response* | *MYLK* |
|  | GO:0042311 | vasodilation | PDE5A,APOE |
|  | *GO:0007596* | *blood coagulation* | *PDE5A, SLC8A1, VWF, PDE3A* |
|  | *GO:0055118* | *negative regulation of cardiac muscle contraction* | *PDE5A* |
|  | *GO:0030168* | *platelet activation* | *PDE5A, SLC8A1, VWF, PDE3A* |
|  | *GO:0007599* | *hemostasis* | *VWF* |
|  | *GO:0010613* | *positive regulation of cardiac muscle hypertrophy* | *PDE5A* |
|  | *GO:0043117* | *positive regulation of vascular permeability* | *PDE3A* |
|  | *GO:0043116* | *negative regulation of vascular permeability* | *PDE3A* |
|  | *GO:0010544* | *negative regulation of platelet activation* | *APOE* |
|  | *GO:0003093* | *regulation of glomerular filtration* | *PTPRO* |
|  | GO:0042742 | defense response to bacterium | DEFB114, DEFB133 |
|  | GO:0051005 | negative regulation of lipoprotein lipase activity | APOC1 |
|  | GO:0048070 | regulation of developmental pigmentation | ADAMTS9 |
|  | *GO:0045636* | *positive regulation of melanocyte differentiation* | *ADAMTS9* |
|  | GO:0005513 | detection of calcium ion | SYT1 |
|  | *GO:0000302* | *response to reactive oxygen species* | *APOE* |
|  | GO:0006516 | glycoprotein catabolic process | ADAMTS9 |
|  | *GO:0034205* | *beta-amyloid formation* | *PION* |
|  | GO:0046086 | adenosine biosynthetic process | NT5E |
|  | GO:0051260 | protein homooligomerization | SYT1, VWF |
|  | *GO:0032463* | *negative regulation of protein homooligomerization* | *CRBN* |
|  | GO:0051651 | maintenance of location in cell | APOE |
|  | GO:0010873 | positive regulation of cholesterol esterification | APOC1, APOE |
|  | *GO:0045833* | *negative regulation of lipid metabolic process* | *APOC1* |
|  | *GO:0008203* | *cholesterol metabolic process* | *APOC1, APOE* |
|  | *GO:0010900* | *negative regulation of phosphatidylcholine catabolic process* | *APOC1* |
|  | *GO:0045541* | *negative regulation of cholesterol biosynthetic process* | *APOE* |
|  | *GO:0006704* | *glucocorticoid biosynthetic process* | *HSD11B1* |
|  | *GO:0045717* | *negative regulation of fatty acid biosynthetic process* | *APOC1* |
|  | *GO:0046839* | *phospholipid dephosphorylation* | *PPAPDC1A* |
|  | GO:0045750 | positive regulation of S phase of mitotic cell cycle | SMAD6 |
|  | *GO:0007128* | *meiotic prophase I* | *SYCP2* |
|  | *GO:0040020* | *regulation of meiosis* | *PDE3A* |
|  | GO:0032805 | positive regulation of low-density lipoprotein particle receptor catabolic process | APOE |
|  | GO:0006874 | cellular calcium ion homeostasis | SLC8A1, APOE |
|  | GO:0060267 | positive regulation of respiratory burst | CAMK1D |
|  | GO:0034766 | negative regulation of ion transmembrane transport | CRBN |
|  | *GO:0048261* | *negative regulation of receptor-mediated endocytosis* | *APOC1* |
|  | *GO:0017158* | *regulation of calcium ion-dependent exocytosis* | *SYT1* |
|  | *GO:0043271* | *negative regulation of ion transport* | *BEST3* |
|  | GO:0014820 | tonic smooth muscle contraction | MYLK |
|  | *GO:0055119* | *relaxation of cardiac muscle* | *PDE5A* |
|  | GO:0010430 | fatty acid omega-oxidation | CYP4V2 |
|  | GO:0071622 | regulation of granulocyte chemotaxis | CAMK1D |
|  | *GO:0007626* | *locomotory behavior* | *OPRM1, NPAS3* |
|  | *GO:0007614* | *short-term memory* | *PDE5A* |
|  | *GO:0002678* | *positive regulation of chronic inflammatory response* | *PDE5A* |
|  | *GO:0050728* | *negative regulation of inflammatory response* | *NT5E, APOE* |
|  | *GO:0042711* | *maternal behavior* | *NPAS3* |
|  | *GO:0030853* | *negative regulation of granulocyte differentiation* | *PRDM16* |
|  | GO:0072358 | cardiovascular system development | APOE |
|  | *GO:0090303* | *positive regulation of wound healing* | *MYLK* |
|  | *GO:0060414* | *aorta smooth muscle tissue morphogenesis* | *MYLK* |
|  | *GO:0007399* | *nervous system development* | *PDE5A, DAB1, CNTN4, CAMK1D, RAB23* |
|  | *GO:0007409* | *axonogenesis* | *PARD3, CNTN4* |
|  | *GO:0008038* | *neuron recognition* | *NTM* |
|  | *GO:0055001* | *muscle cell development* | *SGCZ* |
|  | *GO:0030516* | *regulation of axon extension* | *APOE* |
|  | *GO:0050773* | *regulation of dendrite development* | *CAMK1D* |
|  | *GO:0043586* | *tongue development* | *PRDM16* |
|  | *GO:0060596* | *mammary placode formation* | *NRG3* |
|  | *GO:0014012* | *peripheral nervous system axon regeneration* | *APOE* |
| **MF** | GO:0005516 | calmodulin binding | SLC8A1, SYT1, MYO1B, MYLK, CAMK1D |
|  | GO:0008081 | phosphoric diester hydrolase activity | PDE5A, PDE3A |
|  | *GO:0004331* | *fructose-2,6-bisphosphate 2-phosphatase activity* | *PFKFB3* |
|  | GO:0015204 | urea transmembrane transporter activity | SLC14A2 |
|  | GO:0030617 | transforming growth factor beta receptor, inhibitory cytoplasmic mediator activity | SMAD6 |
|  | GO:0060228 | phosphatidylcholine-sterol O-acyltransferase activator activity | APOC1, APOE |
|  | GO:0070524 | 11-beta-hydroxysteroid dehydrogenase (NADP+) activity | HSD11B1 |
|  | *GO:0003845* | *11-beta-hydroxysteroid dehydrogenase [NAD(P)] activity* | *HSD11B1* |
|  | GO:0004687 | myosin light chain kinase activity | MYLK |
|  | GO:0046911 | metal chelating activity | APOE |
|  | GO:0071208 | histone pre-mRNA DCP binding | SNRPD3 |
|  | GO:0071813 | lipoprotein particle binding | APOE |
|  | GO:0005543 | phospholipid binding | SYT1, SYT14, APOE |
|  | *GO:0005547* | *phosphatidylinositol-3,4,5-trisphosphate binding* | *PARD3,MYO1B* |
|  | *GO:0005546* | *phosphatidylinositol-4,5-bisphosphate binding* | *PARD3,MYO1B* |
|  | GO:0016787 | hydrolase activity | PDE5A, PTPRO, GDPD4,NT5E, ABHD12B, PPAPDC1A, PDE3A, PFKFB3, TDRD9 |
|  | GO:0000166 | nucleotide binding | GNAL, PDE5A, ABCA12, MYO1B, NT5E, MYLK, PFKFB3, HSD11B1, CAMK1D, RAB23, CHD9, TDRD9 |
|  | GO:0017113 | dihydropyrimidine dehydrogenase (NADP+) activity | DPYD |
|  | *GO:0004158* | *dihydroorotate oxidase activity* | *DPYD* |
|  | GO:0046872 | metal ion binding | GNAL, PDE5A, BNC2, ZNF787, GDPD4, ADAM12, NT5E, ZC3H3, MYLK, PDE3A, PRDM16, CYP4V2, ADAMTS9, DPYD |
|  | GO:0015085 | calcium ion transmembrane transporter activity | SLC8A1 |
|  | *GO:0005432* | *calcium:sodium antiporter activity* | *SLC8A1* |
|  | GO:0003873 | 6-phosphofructo-2-kinase activity | PFKFB3 |
|  | GO:0001540 | beta-amyloid binding | APOE, PION |
|  | GO:0050750 | low-density lipoprotein particle receptor binding | SYT1, APOE |
|  | *GO:0070326* | *very-low-density lipoprotein particle receptor binding* | *APOE* |
|  | GO:0070698 | type I activin receptor binding | SMAD6 |
|  | GO:0019865 | immunoglobulin binding | VWF |
|  | GO:0055102 | lipase inhibitor activity | APOC1 |
|  | GO:0004119 | cGMP-inhibited cyclic-nucleotide phosphodiesterase activity | PDE3A |
|  | GO:0003779 | actin binding | MYO1B,MYLK,PHACTR3,SNTG1 |
|  | GO:0030297 | transmembrane receptor protein tyrosine kinase activator activity | NRG3 |
| **CC** | GO:0005576 | extracellular region | APOC1, NRG3, DEFB114, ADAM12, VWF, APOE, NPFF, CNTN4, DEFB133, SNED1, EYS, PDZD2, ADAMTS9, ITFG1 |
|  | GO:0005783 | endoplasmic reticulum | APOC1, OPRM1, SEL1L, VWF, HSD11B1, CYP4V2, PDZD2, KIAA0368 |
|  | GO:0016020 | membrane | CSMD1, ABCA12, SLC8A1, TMEM217, SYT1, PTPRO, GDPD4, CRBN, TMEM206, SYT14, UNC5D, SEL1L, TSPAN13, AGPAT4, PPAPDC1A,TOMM40, PDE3A, FER1L5, HSD11B1, CYP4V2, TRAF3IP3, SLC25A21, ITFG1 |
|  | GO:0031225 | anchored to membrane | NT5E, CNTN4, NTM |
|  | GO:0042627 | chylomicron | APOC1, APOE |
|  | *GO:0034364* | *high-density lipoprotein particle* | *APOC1, APOE* |
|  | *GO:0034361* | *very-low-density lipoprotein particle* | *APOC1, APOE* |
|  | GO:0016013 | syntrophin complex | SNTG1 |
|  | GO:0033093 | Weibel-Palade body | VWF |
|  | *GO:0060203* | *clathrin sculpted glutamate transport vesicle membrane* | *SYT1* |
|  | *GO:0070083* | *clathrin sculpted monoamine transport vesicle membrane* | *SYT1* |
|  | GO:0016021 | integral to membrane | CSMD1, ABCA12, OPRM1, SLC8A1, TMEM217, SYT1, PTPRO, SGCZ, GDPD4, TMEM206, SYT14, ADAM12, UNC5D, SEL1L, SLC14A2, AGPAT4, PPAPDC1A, PDE3A, FER1L5, HSD11B1, CYP4V2, TRAF3IP3, BEST3, SLC25A21, ITFG1 |
|  | GO:0005770 | late endosome | APOE, KIAA0368 |
|  | GO:0031232 | extrinsic to external side of plasma membrane | APOE |

*MF:* GO molecular function; *CC:* GO cellular compartment; *BP:* biological process. Italicized entries represent redundant terms; they are placed under their most informative common ancestor (in normal font).

**Table S4.** Significantly overrepresented GO terms related to the genes in the top 200 dataset SNPs in the TGen dataset.

|  | **GO term identifier** | **GO term description** | **Gene Symbol** |
| --- | --- | --- | --- |
| BP | GO:0030516 | regulation of axon extension | APOE,NRCAM |
|  | *GO:0010976* | *positive regulation of neuron projection development* | *DCC,CAMK1D* |
|  | *GO:0045162* | *clustering of voltage-gated sodium channels* | *NRCAM* |
|  | *GO:0090050* | *positive regulation of cell migration involved in sprouting angiogenesis* | *HDAC9* |
|  | *GO:0007409* | *axonogenesis* | *DCC,NRCAM* |
|  | *GO:0003007* | *heart morphogenesis* | *DLC1,COL11A1* |
|  | *GO:0035767* | *endothelial cell chemotaxis* | *PRKD1* |
|  | *GO:0045823* | *positive regulation of heart contraction* | *GLP1R* |
|  | *GO:0072358* | *cardiovascular system development* | *APOE* |
|  | *GO:0045666* | *positive regulation of neuron differentiation* | *NRCAM,VWC2* |
|  | *GO:0048842* | *positive regulation of axon extension involved in axon guidance* | *DSCAM* |
|  | *GO:0045617* | *negative regulation of keratinocyte differentiation* | *TP63* |
|  | *GO:0002021* | *response to dietary excess* | *APOE* |
|  | *GO:0060513* | *prostatic bud formation* | *TP63* |
|  | *GO:0045597* | *positive regulation of cell differentiation* | *GLP1R,SMAD9* |
|  | *GO:0060529* | *squamous basal epithelial stem cell differentiation involved in prostate gland acinus development* | *TP63* |
|  | *GO:0050773* | *regulation of dendrite development* | *CAMK1D* |
|  | *GO:0002064* | *epithelial cell development* | *TP63* |
|  | *GO:0002063* | *chondrocyte development* | *COL11A1* |
|  | *GO:0001736* | *establishment of planar polarity* | *TP63* |
|  | *GO:0021575* | *hindbrain morphogenesis* | *DLC1* |
|  | *GO:2000171* | *negative regulation of dendrite development* | *DCC* |
|  | *GO:0048671* | *negative regulation of collateral sprouting* | *DCC* |
|  | *GO:0014706* | *striated muscle tissue development* | *EYA2* |
|  | *GO:0070593* | *dendrite self-avoidance* | *DSCAM* |
|  | *GO:0043589* | *skin morphogenesis* | *TP63* |
|  | *GO:0051153* | *regulation of striated muscle cell differentiation* | *HDAC9* |
|  | *GO:0014012* | *peripheral nervous system axon regeneration* | *APOE* |
|  | *GO:0048742* | *regulation of skeletal muscle fiber development* | *HDAC9* |
|  | *GO:0030859* | *polarized epithelial cell differentiation* | *TP63* |
|  | GO:0033700 | phospholipid efflux | APOC1,APOE |
|  | *GO:0033344* | *cholesterol efflux* | *APOC1,APOE* |
|  | *GO:0032368* | *regulation of lipid transport* | *APOC1* |
|  | *GO:0032374* | *regulation of cholesterol transport* | *APOC1* |
|  | *GO:0032375* | *negative regulation of cholesterol transport* | *APOC1* |
|  | GO:0051410 | detoxification of nitrogen compound | MOSC1 |
|  | GO:0030029 | actin filament-based process | ELMO1 |
|  | GO:0042157 | lipoprotein metabolic process | APOC1,APOE |
|  | GO:0042126 | nitrate metabolic process | MOSC1 |
|  | GO:0016266 | O-glycan processing | GXYLT2 |
|  | *GO:0006029* | *proteoglycan metabolic process* | *COL11A1* |
|  | GO:0042159 | lipoprotein catabolic process | APOE |
|  | *GO:0042158* | *lipoprotein biosynthetic process* | *APOE* |
|  | GO:0046717 | acid secretion | SLC22A16 |
|  | GO:0010873 | positive regulation of cholesterol esterification | APOC1,APOE |
|  | *GO:0042981* | *regulation of apoptotic process* | *DCC,CAMK1D,ALK* |
|  | *GO:0045833* | *negative regulation of lipid metabolic process* | *APOC1* |
|  | *GO:0008203* | *cholesterol metabolic process* | *APOC1,APOE* |
|  | *GO:2000107* | *negative regulation of leukocyte apoptotic process* | *CXCL12* |
|  | *GO:0045541* | *negative regulation of cholesterol biosynthetic process* | *APOE* |
|  | *GO:0043523* | *regulation of neuron apoptotic process* | *TP63* |
|  | *GO:0006917* | *induction of apoptosis* | *DCC,DLC1,APOE,TP63* |
|  | *GO:0045717* | *negative regulation of fatty acid biosynthetic process* | *APOC1* |
|  | GO:0032793 | positive regulation of CREB transcription factor activity | PRKD1,CAMK1D |
|  | *GO:0060158* | *activation of phospholipase C activity by dopamine receptor signaling pathway* | *GNA14* |
|  | *GO:0051005* | *negative regulation of lipoprotein lipase activity* | *APOC1* |
|  | *GO:0007202* | *activation of phospholipase C activity* | *DLC1,PDE1C* |
|  | GO:0055085 | transmembrane transport | SLC22A16,KCNH7,HK1,RYR3,ATP6V1C2,SLCO3A1 |
|  | GO:0046069 | cGMP catabolic process | PDE1C |
|  | *GO:0030828* | *positive regulation of cGMP biosynthetic process* | *APOE* |
|  | GO:0007186 | G-protein coupled receptor signaling pathway | GABBR2,ENPP2,APOE,CXCL12,GNA14 |
|  | *GO:0007165* | *signal transduction* | *DLC1,PRKD1,PDE1C,ARHGAP23,ALK,CXCL12,PRKG1,GNA14* |
|  | GO:0006396 | RNA processing | CELF2,RCL1 |
|  | GO:0030203 | glycosaminoglycan metabolic process | CHST9 |
|  | GO:0034638 | phosphatidylcholine catabolic process | ENPP2 |
|  | *GO:0006641* | *triglyceride metabolic process* | *APOC1,APOE* |
|  | *GO:0010900* | *negative regulation of phosphatidylcholine catabolic process* | *APOC1* |
|  | GO:0071205 | protein localization to juxtaparanode region of axon | NFASC |
|  | GO:0032581 | ER-dependent peroxisome organization | PEX16 |
|  | *GO:0016557* | *peroxisome membrane biogenesis* | *PEX16* |
|  | *GO:0016558* | *protein import into peroxisome matrix* | *PEX16* |
|  | *GO:0006625* | *protein targeting to peroxisome* | *PEX16* |
|  | *GO:0045046* | *protein import into peroxisome membrane* | *PEX16* |
|  | GO:0015879 | carnitine transport | SLC22A16 |
|  | GO:0044245 | polysaccharide digestion | SI |
|  | GO:0019934 | cGMP-mediated signaling | APOE |
|  | GO:0032488 | Cdc42 protein signal transduction | APOE |
|  | *GO:0035024* | *negative regulation of Rho protein signal transduction* | *DLC1* |
|  | *GO:0016601* | *Rac protein signal transduction* | *ELMO1* |
|  | GO:0007499 | ectoderm and mesoderm interaction | TP63 |
|  | *GO:0007501* | *mesodermal cell fate specification* | *EYA2* |
|  | GO:0006376 | mRNA splice site selection | CELF2 |
|  | GO:0034447 | very-low-density lipoprotein particle clearance | APOC1,APOE |
|  | *GO:0034375* | *high-density lipoprotein particle remodeling* | *APOC1,APOE* |
|  | *GO:0034380* | *high-density lipoprotein particle assembly* | *APOE* |
|  | *GO:0034379* | *very-low-density lipoprotein particle assembly* | *APOC1* |
|  | *GO:0034384* | *high-density lipoprotein particle clearance* | *APOE* |
|  | *GO:0034382* | *chylomicron remnant clearance* | *APOC1,APOE* |
|  | *GO:0034372* | *very-low-density lipoprotein particle remodeling* | *APOE* |
|  | *GO:0034369* | *plasma lipoprotein particle remodeling* | *APOC1* |
|  | *GO:0010916* | *negative regulation of very-low-density lipoprotein particle clearance* | *APOC1* |
|  | GO:0002175 | protein localization to paranode region of axon | NFASC |
|  | GO:0000173 | inactivation of MAPK activity involved in osmosensory signaling pathway | MBIP |
|  | GO:0050910 | detection of mechanical stimulus involved in sensory perception of sound | COL11A1 |
|  | GO:0071622 | regulation of granulocyte chemotaxis | CAMK1D |
|  | *GO:0009750* | *response to fructose stimulus* | *SI* |
|  | *GO:0009744* | *response to sucrose stimulus* | *SI* |
|  | *GO:0090331* | *negative regulation of platelet aggregation* | *PRKG1* |
|  | *GO:0048261* | *negative regulation of receptor-mediated endocytosis* | *APOC1* |
|  | *GO:0090026* | *positive regulation of monocyte chemotaxis* | *CXCL12* |
|  | *GO:0032869* | *cellular response to insulin stimulus* | *HDAC9,DLC1* |
|  | *GO:0045806* | *negative regulation of endocytosis* | *PRKD1* |
|  | *GO:0051929* | *positive regulation of calcium ion transport via voltage-gated calcium channel activity* | *GLP1R* |
|  | *GO:0070098* | *chemokine-mediated signaling pathway* | *CXCL12* |
|  | *GO:0010544* | *negative regulation of platelet activation* | *APOE* |
|  | *GO:0006911* | *phagocytosis, engulfment* | *ELMO1* |
|  | GO:0031571 | mitotic cell cycle G1/S transition DNA damage checkpoint | TP63 |
|  | GO:0000302 | response to reactive oxygen species | APOE |
|  | GO:0006874 | cellular calcium ion homeostasis | RYR3,APOE,CXCL12 |
|  | GO:0030913 | *paranodal junction assembly* | *NFASC* |
|  | GO:0022011 | *myelination in peripheral nervous system* | *ARHGEF10* |
|  | GO:0050658 | RNA transport | RBFOX1 |
|  | GO:0060060 | post-embryonic retina morphogenesis in camera-type eye | DSCAM |
|  | GO:0048807 | *female genitalia morphogenesis* | *TP63* |
|  | GO:0001880 | *Mullerian duct regression* | *SMAD9* |
|  | GO:0032805 | positive regulation of low-density lipoprotein particle receptor catabolic process | APOE |
|  | *GO:0035307* | *positive regulation of protein dephosphorylation* | *DLC1* |
|  | *GO:0033126* | *positive regulation of GTP catabolic process* | *ARHGEF10* |
|  | GO:0030036 | actin cytoskeleton organization | DLC1,ELMO1,PRKG1 |
|  | *GO:0090307* | *spindle assembly involved in mitosis* | *ARHGEF10* |
|  | *GO:0051497* | *negative regulation of stress fiber assembly* | *DLC1* |
|  | *GO:0016568* | *chromatin modification* | *HDAC9,HIRA,EYA2* |
|  | *GO:0008064* | *regulation of actin polymerization or depolymerization* | *CXCL12* |
|  | GO:0060197 | cloacal septation | TP63 |
|  | *GO:0060157* | *urinary bladder development* | *TP63* |
|  | *GO:0030850* | *prostate gland development* | *TP63* |
|  | GO:0051651 | maintenance of location in cell | APOE |
|  | GO:2001044 | regulation of integrin-mediated signaling pathway | PRKD1 |
|  | GO:0060267 | positive regulation of respiratory burst | CAMK1D |
|  | GO:0010468 | regulation of gene expression | APOE,PRDM14 |
|  | GO:0016576 | histone dephosphorylation | EYA2 |
|  | *GO:0034983* | *peptidyl-lysine deacetylation* | *HDAC9* |
|  | *GO:0070932* | *histone H3 deacetylation* | *HDAC9* |
|  | *GO:0070933* | *histone H4 deacetylation* | *HDAC9* |
| MF | GO:0004551 | nucleotide diphosphatase activity | ENPP2 |
|  | GO:0005275 | amine transmembrane transporter activity | SLC22A16 |
|  | GO:0008528 | G-protein coupled peptide receptor activity | GLP1R |
|  | GO:0060228 | phosphatidylcholine-sterol O-acyltransferase activator activity | APOC1,APOE |
|  | GO:0071813 | lipoprotein particle binding | APOE |
|  | GO:0001537 | N-acetylgalactosamine 4-O-sulfotransferase activity | CHST9 |
|  | GO:0003963 | RNA-3'-phosphate cyclase activity | RCL1 |
|  | GO:0003997 | acyl-CoA oxidase activity | ACOXL |
|  | GO:0070326 | very-low-density lipoprotein particle receptor binding | APOE |
|  | GO:0030151 | molybdenum ion binding | MOSC1 |
|  | GO:0043546 | molybdopterin cofactor binding | MOSC1 |
|  | GO:0000166 | nucleotide binding | CELF2,KIF5C,HK1,PRKD1,RAB3C,RBM20,CAMK1D,ALK,KIAA0564,RBFOX1,C8orf80,PRKG1,GNA14 |
|  | GO:0004438 | phosphatidylinositol-3-phosphatase activity | MTMR14 |
|  | GO:0016763 | transferase activity, transferring pentosyl groups | GXYLT2 |
|  | GO:0004558 | alpha-glucosidase activity | SI |
|  | GO:0008940 | nitrate reductase activity | MOSC1 |
|  | GO:0004396 | hexokinase activity | HK1 |
|  | GO:0048156 | tau protein binding | APOE |
|  | GO:0030618 | transforming growth factor beta receptor, pathway-specific cytoplasmic mediator activity | SMAD9 |
|  | GO:0015278 | calcium-release channel activity | RYR3 |
|  | GO:0004692 | cGMP-dependent protein kinase activity | PRKG1 |
|  | GO:0034190 | apolipoprotein receptor binding | KIF5C |
|  | GO:0004528 | phosphodiesterase I activity | ENPP2 |
|  | GO:0045236 | CXCR chemokine receptor binding | CXCL12 |
|  | GO:0055102 | lipase inhibitor activity | APOC1 |
|  | GO:0048101 | calcium- and calmodulin-regulated 3',5'-cyclic-GMP phosphodiesterase activity | PDE1C |
|  | GO:0005042 | netrin receptor activity | DCC |
|  | GO:0035252 | UDP-xylosyltransferase activity | GXYLT2 |
|  | GO:0004965 | G-protein coupled GABA receptor activity | GABBR2 |
| CC | GO:0005576 | extracellular region | APOC1,ENPP2,CHST9,APOE,DSCAM,IGSF21,KIAA0564,COL11A1,CXCL12,VWC2 |
|  | GO:0033010 | paranodal junction | NFASC |
|  | GO:0042627 | chylomicron | APOC1,APOE |
|  | *GO:0034363* | *intermediate-density lipoprotein particle* | *APOE* |
|  | *GO:0034364* | *high-density lipoprotein particle* | *APOC1,APOE* |
|  | *GO:0034361* | *very-low-density lipoprotein particle* | *APOC1,APOE* |
|  | GO:0043005 | neuron projection | GABBR2,KIF5C,NRCAM |
|  | GO:0045121 | membrane raft | DCC,DLC1,HK1,SI |
|  | GO:0005829 | cytosol | DCC,DLC1,HK1,ARHGEF10,PRKD1,ELMO1,RAB3C,ATP6V1C2,TP63,PDE1C,SMAD9,PRKG1 |
|  | GO:0005622 | intracellular | ZNF407,DLC1,ARHGEF10,PSD3,PRDM14,RBM20,ATF7,ARHGAP23,SMAD9,STAU2 |
|  | GO:0045211 | postsynaptic membrane | GABBR2,DLGAP1,PSD3 |
|  | GO:0005887 | integral to plasma membrane | GABBR2,GLP1R,ENPP2,PRKD1,DSCAM,ALK,NRCAM |
|  | GO:0030314 | junctional membrane complex | RYR3 |
|  | GO:0045334 | clathrin-coated endocytic vesicle | GLP1R |
|  | GO:0005954 | calcium- and calmodulin-dependent protein kinase complex | CAMK1D |
|  | GO:0005634 | nucleus | HDAC9,CELF2,ZNF407,DLC1,HK1,PRKD1,PRDM14,MBIP,RBM20,ATF7,CAMK1D,TP63,HIRA,RCL1,SMAD9,RBFOX1,C8orf80,STAU2,EYA2 |
|  | GO:0005737 | cytoplasm | GABBR2,MTMR14,HDAC9,CELF2,KIF5C,DLC1,PRKD1,PSD3,ELMO1,APOE,MBIP,RAB3C,ATF7,CAMK1D,TP63,SMAD9,RBFOX1,PRKG1,STAU2,MTUS2,EYA2 |
|  | GO:0005592 | collagen type XI | COL11A1 |
|  | GO:0034399 | nuclear periphery | ATF7 |
|  | GO:0016021 | integral to membrane | DCC,NFASC,GLP1R,SLC22A16,KCNH7,RYR3,PEX16,CHST9,THSD7A,TM4SF20,GXYLT2,LINGO2,TMEM132D,FNDC3B,SLC35F3,SI,SLCO3A1 |
|  | GO:0005874 | microtubule | KIF5C,APOE,STAU2 |
|  | GO:0033270 | paranode region of axon | NFASC |
|  | GO:0033268 | *node of Ranvier* | *NFASC* |
|  | GO:0005886 | plasma membrane | DCC,GABBR2,DLGAP1,NFASC,GLP1R,ENPP2,SLC22A16,KCNH7,PRKD1,PSD3,ELMO1,APOE,RAB3C,TM4SF20,DSCAM,ALK,PRKG1,NRCAM,SI,GNA14,SLCO3A1 |
|  | GO:0031232 | extrinsic to external side of plasma membrane | APOE |
|  | GO:0032584 | growth cone membrane | DCC |
|  | GO:0005667 | transcription factor complex | HDAC9,TP63,SMAD9 |

*MF:* GO molecular function; *CC:* GO cellular compartment; *BP:* biological process, *No:* number of genes from the list that have the relevant annotation. . Italicized entries represent the redundant terms; they are placed under their most informative common ancestor (in normal font).
